# Expression and Localization of CLC Chloride Transport Proteins in the Avian Retina

**DOI:** 10.1371/journal.pone.0017647

**Published:** 2011-03-07

**Authors:** Emily McMains, Vijai Krishnan, Sujitha Prasad, Evanna Gleason

**Affiliations:** Department of Biological Sciences, Louisiana State University, Baton Rouge, Louisiana, United States of America; Flinders University, Australia

## Abstract

Members of the ubiquitously expressed CLC protein family of chloride channels and transporters play important roles in regulating cellular chloride and pH. The CLCs that function as Cl^−^/H^+^ antiporters, ClCs 3–7, are essential in particular for the acidification of endosomal compartments and protein degradation. These proteins are broadly expressed in the nervous system, and mutations that disrupt their expression are responsible for several human genetic diseases. Furthermore, knock-out of ClC3 and ClC7 in the mouse result in the degeneration of the hippocampus and the retina. Despite this evidence of their importance in retinal function, the expression patterns of different CLC transporters in different retinal cell types are as yet undescribed. Previous work in our lab has shown that in chicken amacrine cells, internal Cl^−^ can be dynamic. To determine whether CLCs have the potential to participate, we used PCR and immunohistochemical techniques to examine CLC transporter expression in the chicken retina. We observed a high level of variation in the retinal expression levels and patterns among the different CLC proteins examined. These findings, which represent the first systematic investigation of CLC transporter expression in the retina, support diverse functions for the different CLCs in this tissue.

## Introduction

The distribution of Cl^−^ across the plasma membrane is known to be tightly regulated, primarily by the activity of two Cl^−^ co-transport proteins: the potassium/chloride co-transporter (KCC) and the sodium/potassium/chloride co-transporter (NKCC). These transport proteins are known to play a critical role in setting the equilibrium potential for Cl^−^ by adjusting cytosolic Cl^−^ concentrations and thus determining the sign (inhibitory or excitatory) of synaptic transmission mediated by GABA- or glycine-gated Cl^−^ channels. Another group of Cl^−^ transporters, the CLC transporters, also move Cl^−^ across cellular membranes. A subset of these transporters (ClCs 3–7) are chiefly expressed on intracellular membranes (for review, see [1]). The activity of these transporters also has the potential to affect Cl^−^ distribution and may prove to be another important factor contributing to the dynamic nature of cytosolic Cl^−^ concentration [2].

The vesicular CLCs can be subdivided into two groups based on sequence similarity: ClCs 3, 4, 5 and ClCs 6, 7. Over the last several years, biophysical studies of these transporters have established that ClCs 3–7 are all Cl^−^/H^+^ antiporters [3], [4], [5], [6], [7], [8]. Furthermore, it has been established for ClC 4, 5 and 7 that the stoichiometry of exchange is 2Cl^−^∶1H^+^ [3], [4], [5]. Additionally, CLCs are thought to operate as dimers with both homo- and heterodimers observed in expression systems [9], [10]. While the biophysical properties of the vesicular CLCs are becoming clear, the functions of these transporters are less well understood. Endosomal compartments are typically acidic due to the presence of the V-type proton pump. This acidic environment can facilitate other transport mechanisms and provide optimal conditions for some enzyme functions. One proposed role for the internal CLCs is that they move Cl^−^ into endosomes as a counter ion to relieve the steep membrane potential generated by the proton pumping [11]. The cost of this CLC-dependent Cl^−^ transport, however, is the exchange for protons previously moved into the compartment by the ATP-dependent proton pump. The resolution of this dilemma may be emerging from recent studies utilizing mutations that convert ClCs 5 and 7 from Cl^−^/H^+^ antiporters into pure Cl^−^ conductors [12], [13]. The results of these studies imply that the primary function of some CLCs may be to concentrate Cl^−^ in endosomal compartments. Another recent study provides evidence that ClC5 plays a direct role in endosomal acidification itself [2]. Thus the roles of the internal CLCs are just coming into focus and it is possible that their functions are diverse and may be dependent upon the specific cellular context in which they reside.

The expression of ClCs 3–7 varies among cell types, however, expression has been demonstrated for each of these transporters in neuronal tissues [14], [15], [16], [17], [18]. Within cells, however, their distribution differs with respect to their position in the endosomal pathway. ClC3 resides in mid to late endosomes and synaptic vesicles [19]. The localization of ClC4 is not completely resolved but it seems to be in endosomal rather than lysosomal compartments [1]. ClC5 is typically found in early endosomes [20], and ClCs 6 and 7 in late endosomes and lysosomes [15], [17]. Knockout studies and mutational analyses have shed light on some of the physiological consequences of impaired CLC function. ClC5 is mutated in the human kidney disorder Dent's disease [21], and ClC5 knockouts display impairments in the endocytic pathway [22]. ClC7 knockouts developed osteopetrosis and lysosomal storage disease in the central nervous system and in the kidney [15], [23]. Furthermore, knockouts in ClCs 3 and 7 led to loss of hippocampal tissue and especially relevant to this work, marked retinal degeneration [19], [23]. The dramatic effects of these knockouts on retinal tissue suggest the hypothesis that these transporters are critical to normal retinal function and this possibility is one motivation for our investigation into the retinal localization of the vesicular CLCs. We are also motivated to learn more about the vesicular CLCs in the retina because we have previously demonstrated that nitric oxide (NO) causes the release of Cl^−^ from an internal store in cultured avian amacrine cells and rat hippocampal neurons [24]. A functional link between the CLCs and the effects of NO is yet to be established but the ability of these transporters to load Cl^−^ into a potentially releasable store compels us to examine their retinal expression pattern. Furthermore, despite the important role of certain CLC family members in normal retinal development suggested by analysis of knock-out mice [19], [23], no systematic examination of CLC transporter expression has yet been carried out in the retina of any species. Here we explore the expression and localization of ClCs 3,4,5,6 and 7 in the adult avian retina using polyclonal antibodies raised against each of these transporters. Double-labeling with retinal cell markers is used to further define the cellular expression patterns for each of the proteins. We find that each of the CLCs has a unique but sometimes overlapping expression pattern in the avian retina. Furthermore, the CLC-specific labeling patterns we describe are due to differences in CLC expression among cell types as well as among sub-cellular compartments. The specificity of CLC localization implies that these transporters subserve specific and potentially diverse functions in the retina.

## Materials and Methods

### Ethics Statement

All methods using animals in this study were approved by the Institutional Animal Care and Use Committee, Louisiana State University (Assurance #A3612-01).

### PCR amplification

Expression of mRNAs coding for CLCs 3–7 was studied using the reverse transcription-polymerase chain reaction method. A mixed population of cultured chick retinal cells (embryonic equivalent day 15) was collected in 100 µl lysis buffer+5 µl of 0.1 µM stock dithiothreitol and 40 units (1 µl) of RNAase inhibitor (Invitrogen, Carlsbad, CA). Isolation of mRNA was performed using dynabeads mRNA DIRECT Micro kit (Invitrogen). cDNA synthesis and PCR amplification was conducted in a single tube using a one-step RT-PCR system which includes a Superscript III one-step RT-PCR mix consisting of both platinum Taq polymerase and reverse transcriptase (Invitrogen). Two sets of gene specific primers (Table 1) were designed for each CLC type and used at a 0.5 µM concentration in the PCR reaction mixture. Efficient cDNA synthesis was achieved with a 30 min incubation at 55°C for CLCs 3,4,5 and 7 and the same incubation time of 30 min at a temperature of 50°C for CLC-6. Amplification of the reverse-transcribed cDNA was performed using a Bio-Rad C-1000 thermocycler (Hercules, CA). An initial denaturation was conducted at 94°C for 2 min. Forty cycles of PCR were carried out under the following conditions: Denaturing at 94°C for 15 s, annealing at 55°C (CLC-3, 4, 5, and 7) and 50°C (CLC-6) for 30 s and extension at 68°C for 1 min. A final extension was performed at 68°C for 5 min. The predicted sizes of the PCR products are listed in Table 1. The PCR products were run on a 2% agarose gel and stained with ethidium bromide. The gel was visualized with the Bio-Rad gel documentation system. Specificity of PCR products was confirmed through sequencing analysis.

**Table 1:** Primer sequences for PCR amplifications.

Primer	Sequence	Product size (bp)	Annealing temperature
CLC3 forward	5′- GGTGAAGGTGTTTGCTCCAT-3′	187 bp	55°
CLC3 reverse	5′-GCAGGCAACATGTACCAATG-3′	187 bp	55°
CLC3 forward	5′-ATGGTCAGGATGGCTCGTAG-3′	209 bp	55°
CLC3 reverse	5′-CTGCCCAAGTTTTCCACTGT-3′	209 bp	55°
CLC4 forward	5′-GGAGTACCATACCCCCTGGT-3′	231 bp	55°
CLC4 reverse	5′-ATCAGCTCACTGGTGCTCCT-3′	231 bp	55°
CLC4 forward	5′-TCAAAAGACTCGGAGCGACT-3′	226 bp	55°
CLC4 reverse	5′-CCACAGTCTCCATTGGTGTG-3′	226 bp	55°
CLC5 forward	5′-AGGAACCACTTCCTGGTGTG-3′	200 bp	55°
CLC5 reverse	5′-TAAGGAACCTGCCAACAACC-3′	200 bp	55°
CLC5 forward	5′-AGGAACCACTTCCTGGTGTG-3′	235 bp	55°
CLC5 reverse	5′-GTCATCCAGTGGGCAGAAAT-3′	235 bp	55°
CLC6 forward	5′-GGAGTGCCTCTTCTGGAGTG-3′	155 bp	50°
CLC6 reverse	5′-ACAGGGAAGGCATGATGAAC-3′	155 bp	50°
CLC6 forward	5′-TGGAGTATGCAGGGCATGTCAGA-3′	553 bp	50°
CLC6 reverse	5′- TTTCCACTGTTTCCCACTCCA-3′	553 bp	50°
CLC7 forward	5′-TACCGTGTGGTGAAGGACAA-3′	199 bp	55°
CLC7 reverse	5′-CAACATGTGGGATCTTCACG-3′	199 bp	55°
CLC7 forward	5′-TACCGTGTGGTGAAGGACAA-3′	169 bp	55°
CLC7 reverse	5′-AGCATTTGATCTGGGGAATG-3′	169 bp	55°

### Western Blots

Adult White Leghorn chickens were sacrificed by intraperitoneal injection of sodium pentobarbital (250 mg/kg, Sigma-Aldrich, St. Louis, MO) followed by decapitation. Flash-frozen mouse and rat tissue was provided as a gift from Dr. Jacquelyn Stephens, Louisiana State University. Chicken, mouse, and rat brain tissue were homogenized in lysis buffer (2% Triton X-100, 300 mM NaCl, 20 mM Tris, 2 mM EDTA, 2 mM EGTA, 1% NP40) containing a cocktail of protease inhibitors (PMSF (1 mM), leupeptin, (5 µg/mL) aprotinin (2.5 µg/mL), 1,10 ortho-phenantrolin (0.2 µg/mL) and pepstatin (0.7 µg/mL)). Samples were spun at 4,000 rpm for 20 min at 4°C. Protein content was determined using the BCA Protein Assay Kit from Pierce (Rockford, IL). For electrophoresis, protein samples were prepared on ice in loading buffer containing 62.5 mM Tris, 2% SDS, 10% glycerol, 5% B-mercaptoethanol, and 0.005% bromophenol blue. Protein samples were boiled for three minutes prior to loading on the gel. Proteins (50–200 µg) were separated on a 7.5% SDS gel along with 10 µL Pageruler molecular weight markers (Fermatas, Glenburnie, MD), except for blots probed with ClC4 and ClC7 antibody, which were run on 5% SDS gels. Proteins were transferred to nitrocellulose membranes. Membranes were blocked overnight at 4°C in 4% milk and 5% normal goat serum (NGS, ClC3 and ClC5), for one hr at room temperature in 4% milk (ClC6 and ClC7), or for one hr at room temperature in 4% milk and 5% NGS (ClC4). Blocking solutions were all made in tris buffered saline with 0.1% Tween 20 (TBS-T). Primary antibodies were diluted in TBS-T with 1% bovine serum albumin (BSA). ClC6 antibody was diluted in 1% BSA TBS-T with 5% NGS. ClC3 and ClC5 antibodies were applied at 1∶500 for one hr at room temperature. ClC4 and ClC6 were applied at 1∶500 overnight at 4°C, and ClC7 antibody was applied at 1∶500 for 90 min at room temperature. Goat anti-rabbit secondary antibody conjugated on horseradish peroxidase (Pierce, Rockford, IL) was diluted from 1∶1000–1∶5000 in TBS-T with 1% milk. Membranes were incubated in secondary antibodies for 1–1.5 hrs at room temperature. Proteins were visualized using the Supersignal Western Pico Reagent (Pierce).

### Immunocytochemistry

Adult White Leghorn chickens were sacrificed by the same methods indicated for Western blots. The eyes were enucleated and hemisected. After removing the vitreous, eye cups were immersed in 4% paraformaldehyde for 15–20 min at room temperature. Following fixation, eye cups were washed three times in PBS+1% Glycine. Central retina was dissected from the eyecup and incubated in 15% sucrose for 30 min, 20% sucrose for 1 hr, and 30% sucrose solution overnight at 4°C. Retinae were infiltrated in a 2∶1 mixture of 30% sucrose and O.C.T. compound (Sakura Finetek, Torrence, CA) for 30 min. Samples were frozen in fresh sucrose/O.C.T. mixture on a slurry of isopentane and dry ice. Sections (12–16 µm) were cut on a Leica CM1850 cryostat (Wetzlar, Germany) and mounted on Superfrost Plus microscope slides (Ted Pella, Redding, CA).

The primary antibodies used in this work are listed in Table 1 along with the source of the antibodies and the dilution at which they were used. The monoclonal antibody to ClC-3 (cat#75-259, clone N258/5) was developed and/or obtained from the UC Davis/NIH NeuroMab Facility, supported by NIH grant U24NS050606 and maintained by the Department of Neurobiology, Physiology and Behavior, College of Biological Sciences, University of California, Davis, CA 95616. In addition to the CLC antibodies included in this table, five other commercially available CLC antibodies were evaluated and rejected for use in this study based on unsatisfactory Western blot results. Polyclonal antibodies raised against peptides corresponding to regions of rat ClC4 and ClC7 c-terminus (Alpha Diagnostics, CLC41-A and CLC71-A) bound multiple bands at incorrect molecular weights for the respective proteins. Polyclonal antibodies specific for internal regions of human ClC3 and ClC5 (Santa Cruz Biotechnology, SC-17572 and SC-22373) also gave nonspecific immunoreactive bands. Finally, a polyclonal antibody raised against a region of human ClC7 n-terminus gave inconsistent Western blot results (Abcam, Cambridge, MA). Antibodies were diluted in dilution solution consisting of phosphate-buffered saline (PBS), 1% BSA, and 0.1% saponin. Sections were blocked in dilution solution containing 10% NGS (Jackson ImmunoResearch Laboratories, West Grove, PA). Primary antibodies were applied for either 72 hrs at 4°C (anti-CLC3–7, anti-nNOS, anti-calretinin, and anti-PKCα), 1 hr at room temperature (anti-glutamine synthetase, anti-HPC-1, anti-parvalbumin, and anti-SV2), or overnight at room temperature (anti-ChAT). Secondary antibodies were applied for 1 hr at room temperature. Goat anti-rabbit antibodies conjugated either to Cy3 (1∶500, Millipore, Billerica, MA) or to Dylight 488 (1∶100, Pierce) were used to localize polyclonal antibodies. Goat anti-mouse antibodies conjugated to Cy3 or DyLight 488 were used to localize the monoclonal antibodies (1∶100).

Labeled sections were viewed on an inverted Leica TCS SP2 Spectral confocal microscope (Leica Microsystems, Wetzlar, Germany) with 40× and 63× oil immersion objectives (1.25 and 1.4 N.A., respectively). DyLight 488 was observed with the 488 nm laser line with emission collection from 502–553 nm. Cy3 was visualized with the 543 laser line with emission collection from 572–672 nm. Images of double-labeled material were collected by sequential scanning to minimize difficulties due to bleed through. Images were acquired using the Leica LCS software package. All images were collected using the same confocal laser intensity. PMT gain was adjusted separately for each antibody except full retinal section images of CLC single-labeled retinae where PMT was kept the same for comparing labeling intensities of the different CLC antibodies. For this reason, some features that are not as clear in these images are more appreciable in later figures. All images of single and double-labeled tissue are maximum projections of twenty z planes except for 40× images of CLC and PKC and CLC and HPC-1 double labeling and some higher magnification images. Single z plane images of these conditions are shown to provide greater resolution of doubly immunoreactive features and are denoted “1z”. All fluorescent confocal images were median-filtered using the Leica confocal software. Figures were assembled using Adobe Photoshop CS3 (Adobe Systems Inc., San Jose, CA).

CLC antibody specificity in tissue sections was assessed by comparing CLC antibody labeling of retinal sections with and without pre-incubation with their respective antigenic peptides. Antigenic peptides were pre-incubated with primary antibody for 1–2 hrs at room temperature, with antibody: peptide ratios of 1∶1(ClC7), 1∶5 (ClC4), and 1∶10 (ClC5 and ClC6). Peptide exposed sections and non-peptide exposed sections were taken from the same retinae and were processed side by side.

IPL depth was determined by dividing the distance of immunoreactive bands from the INL/IPL border by the total width of the IPL as measured from its border with the INL to its border with the GCL, following the method of Fischer et al. [25]. All IPL depth measurements are based on averages of IPL depth from at least three retinae.

## Results

### CLC transporters are expressed at the mRNA level in cultured chick retinal neurons

**Figure 1:**
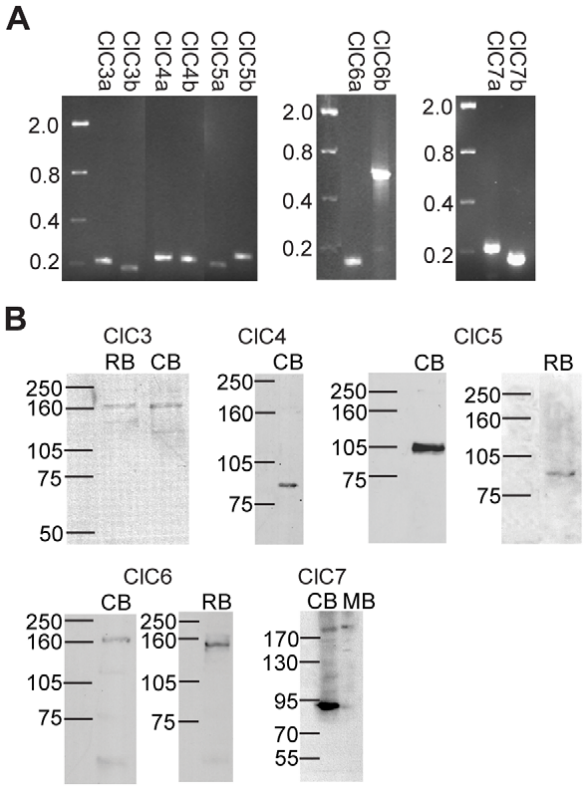
CLC H^+^/Cl^−^ antiporters are expressed in chicken neurons. **A**, RT-PCR amplification of ClCs 3–7 mRNA from a mixed population of cultured chick retinal neurons. Two primer pairs for each transporter amplified single products of the expected size (in kb). **B**, Western blots of chicken, rat, and mouse brain protein probed with antibodies specific for mammalian ClCs 3–7 reveal labeling of bands at the expected molecular weights for each protein. CB, chicken brain; RB, rat brain; MB, mouse brain.

A previous investigation by our group revealed that nitric oxide (NO) induces a transient elevation of cytosolic Cl^−^ in cultured chick amacrine cells and that this increase is due to Cl^−^ release from an internal compartment and not from Cl^−^ crossing the plasma membrane [24]. In an effort to identify candidate intracellular Cl^−^ transporters, PCR amplification of internally-expressed members of the CLC family of Cl^−^ channels and transporters (ClCs 3–7) was conducted from a mixed population of cultured retinal neurons. Primers were designed based on predicted nucleotide sequences for chicken ClC3, ClC4, ClC5, and ClC6 (Table 1). ClC7 primers were derived from the referenced sequence for chicken ClC7 (Caldwell et al, 2005 [26], Table 1). Transcripts for all CLC transporters examined were amplified and verified through sequencing of the PCR product, indicating that the mRNAs encoding these transporters are expressed in cultured chicken retinal cells (Figure 1A).

### CLC antibodies raised against mammalian targets specifically label chicken CLC proteins

To examine CLC expression in the chicken retina, commercially-available antibodies raised against mammalian ClCs 3–7 were obtained. Western blots of chicken brain and retina tissue were probed with these antibodies to assess their specificity for chicken CLCs (Table 2, Fig. 1B). An antibody raised against a region of the rat ClC3 carboxy-terminus labeled a band near 160 kD in blots of chicken and rat brain protein (Fig. 1B). The predicted molecular weight for ClC3 is 96 kD in chicken and 91 kD in rat. All internal CLC transporters are known to form both homo- and hetero-dimers in expression systems [9], [10], [27]. It is possible that the dominant band we observed in both species represents ClC3 dimers. An antibody specific for rat ClC4 revealed a band between 75 and 105 kD which is close to the predicted molecular weight of the chicken ClC4 protein (76 kD). Unfortunately, in our hands, blots of rat and mouse brain homogenates incubated with the same ClC4 antibody showed no reactivity (data not shown). Nonetheless the position of the band in chicken blots was in good agreement with that found in another study of mouse tissue using two different polyclonal antibodies to different regions of mammalian ClC4 protein [28]. Probing rat and chicken brain homogenates with ClC5 antibody revealed a band near 105 kD in the chicken and in between 75 kD and 105 kD in the rat. These values are in line with the respective molecular weights of ClC5 in the two species (104 kD in chicken, 83 kD in rat) and the findings in rat are similar to what has been described by other investigators using different ClC5-specific antibodies [29], [30], [31]. It is important to note that the ClC3, ClC4, and ClC5 antibodies bound different molecular weight proteins despite the sequence similarity of the three proteins, supporting the specificity of each antibody for its respective target. An antibody raised against rat ClC6 labeled a band near 160 kD in blots of chicken and rat brain protein. The expected molecular weight for the protein in both species is 97 kD. It seems likely that the ClC6 antibody bound ClC6 dimers, as was seen for ClC3. Two bands, one faint band slightly above 170 kD and another stronger band between 75 and 90 kD, were labeled in blots of chicken and rat brain protein probed with a ClC7 antibody (expected molecular weights are 88 kD for chicken ClC7 and 89 kD for rat ClC7). The higher bands could represent ClC7 homodimers [27]. It is interesting to note that the intensity of the lower band was much stronger in chicken brain than in rat. Western blot analyses by other groups have described similar results for ClC7 in mammalian systems using different polyclonal antibodies [23]. These results support the expression of ClC3, ClC4, ClC5, ClC6, and ClC7 in chicken brain tissue and, furthermore, confirm the specificity of these mammalian-derived antibodies for these proteins in chicken tissue.

**Table 2:** Antibody Information.

Antibody	Host	Source	Catalog No.	Immunogen	Working Dilution
ClC3	Rabbit, pAb	Alpha Diagnostics	ClC31-A	18 aa synthetic peptide from rat C-terminus	1∶500
ClC3	Mouse, mAb	Neuromab	clone N258/5	Synthetic peptide aa 98–115 derived from rat ClC3 (CKDRERHRRINSKKKESA)	1∶500
ClC4	Rabbit, pAb	Abgent	AP6329f	KLH conjugated synthetic peptide derived from within aa670–695 of human ClC4	1∶500
ClC5	Rabbit, pAb	Alpha Diagnostics	ClC51-A	13 aa synthetic peptide from within rat ClC5 aa 646–676	1∶500
ClC6	Rabbit, pAb	Alpha Diagnostics	ClC61-A	19 aa synthetic peptide from within rat aa 840–870	1∶500
ClC7	Rabbit, pAb	Abcam	Ab31264	Synthetic peptide derived from human ClC7 (RYRLGKRGLEELSLAQT)	1∶500
Calretinin, Clone 6B8.2	Mouse, mAb	Millipore	MAB1568	Recombinant rat calretinin	1∶100
Choline Acetyltransferase, ChAT	Rabbit, pAb	Dr. Miles Epstein	1465	Partially purified ChAT isolated from chicken optic lobes	1∶1000
Glutamine Synthetase, Clone GS-6	Mouse, mAb	Millipore	MAB302	Glutamine synthetase purified from sheep brain	1∶500
Syntaxin 1, Clone HPC-1	Mouse, mAb	Sigma	S0664	Synaptosomal plasma membrane fraction from adult rat hippocampus	1∶100
nNOS, Clone NOS-B1	Mouse, mAb	Sigma	N2280	Recombinant rat brain nNOS, aa 1–181	1∶500
Protein Kinase C, Clone MC5	Mouse, mAb	Abcam	Ab31	Purified bovine brain protein kinase C	1∶100
SV2	Mouse, mAb	Developmental Studies Hybridoma Bank	SV2	Purified synaptic vesicles from Ommata electric organ	supernatant

aa, amino acid.

**Figure 2:**
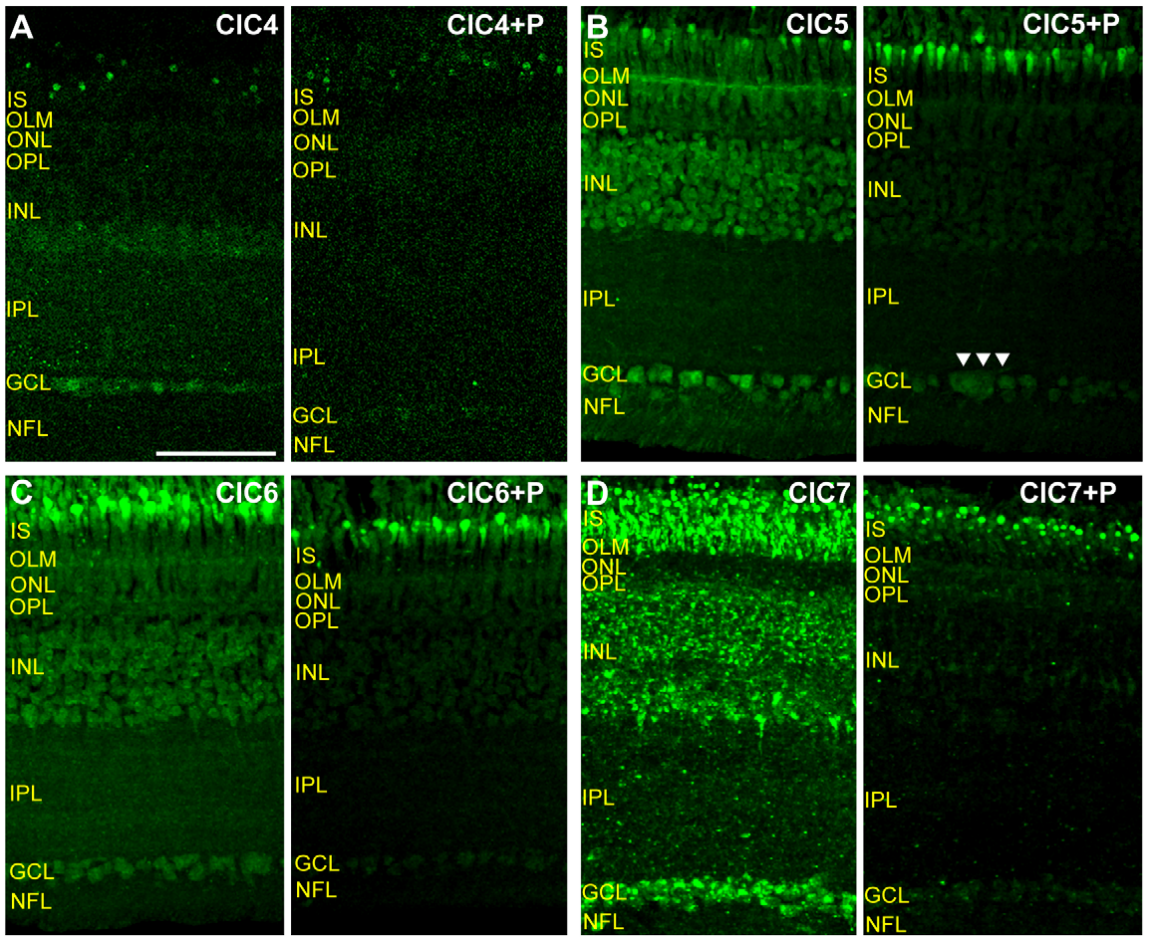
CLC antibodies specifically label CLC proteins in chicken retina. A–D, Images of vertical sections of chicken retina. Each image pair was acquired and processed identically. **A**, **Left**, ClC4 antibody labeling. **Right**, Pre-incubation with ClC4 peptide (P) dramatically reduced antibody signal. **B**, Labeling of retinal section by ClC5 antibody (left) was substantially diminished by exposure to ClC5 peptide (right) in retinal layers, however a slightly disproportionate signal remained in the ganglion cell layer (GCL, arrowheads). **C**, ClC6 antibody labeling in the presence (right) and absence (left) of ClC6 peptide. ClC6 antibody signal was uniformly and substantially decreased by pre-treatment with peptide. **D**, ClC7 antibody labeling in tissue not exposed (left) and exposed (right) to ClC7 demonstrates near total elimination of the antibody signal. (Scale for A is 50 µm and applies for B–D as well.)

**Figure 3:**
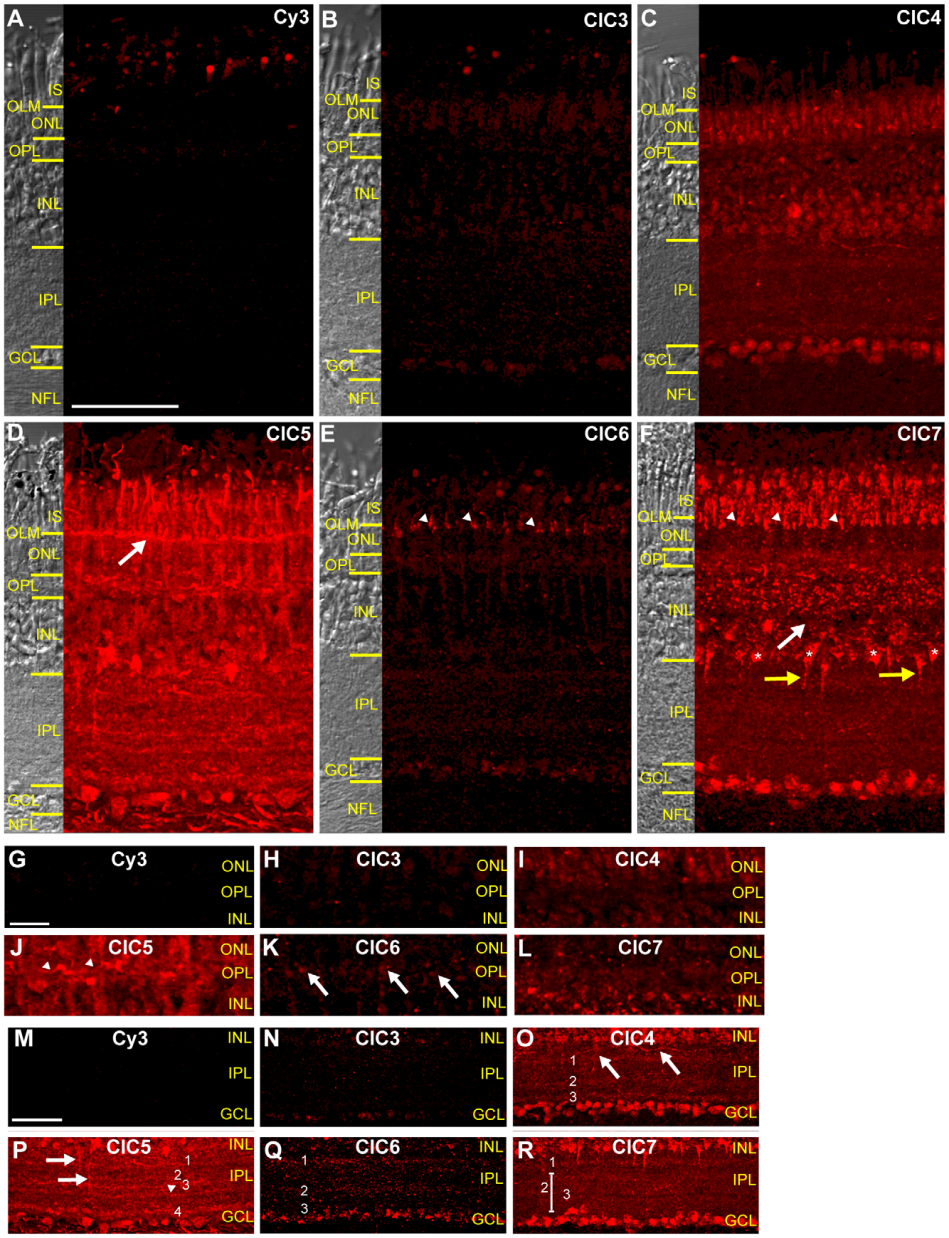
CLC H^+^/Cl^−^ antiporters have diverse expression patterns in mature chicken retina. **A–F**, Images of vertical sections of chicken retina. Images were acquired and processed identically except for F, which was acquired at a lower PMT gain to avoid saturation. **A**, Secondary antibody only-labeled section shows minimal background fluorescence. Signal in photoreceptor outer segments and in oil droplets is autofluorescence. **B**, Labeling of retinal tissue by ClC3 antibody is barely detectable. **C**, The ClC4 antibody labels all three nuclear layers of the retina and the IPL. **D**, ClC5 antibody labeling in nuclear and synaptic layers. Arrow indicates ClC5 expression in the OLM. **E**, The ClC6 antibody labeled vertical elements in photoreceptors (arrowheads). **F**, Punctate ClC7 signal in nuclear and synaptic layers. Asterisks and yellow arrows indicate ClC7+ cells at INL/IPL border. White arrow indicates INL band lacking ClC7. **G–L**, Zoom of CLC antibody labeling in the OPL from the same sections shown in A–F. Images have been individually adjusted to highlight immunoreactive features. ClC5 expression is widespread and diffuse and in scattered processes (arrowheads) whereas ClC6 antibodies label discrete puncta. ClC4 and ClC7 antibody signals are fainter, and ClC3 signal is absent from the OPL. **M–R**, Zoomed images of anti-CLC labeling in the IPL. Images have been individually adjusted. IPL labeling by ClC3 antibody was minimal. ClC4 antibody labels horizontal processes in outer IPL (arrows) and three distinct bands (1–3). ClC5 is expressed in vertical processes projecting to central IPL (arrows) and four (1–4) to five (arrowhead) bands. Three bands of ClC6 immunoreactivity (1–3) are distinguishable. ClC7 antibody labels one band in outer IPL (1) and one band in inner IPL (2). 3 indicates a thin IPL band lacking ClC7. (Scale bar in A is 50 µm and applies to B–F. Scale bar in G is 5 µm and applies to H–L. Scale bar in M is 50 µm and applies to N–R.)

To determine the specificity of the CLC antibodies for CLC proteins in intact chicken retinal tissue, we labeled vertical sections of retina with CLC antibodies that had been pre-incubated with their respective antigenic peptides. Figure 2 shows that, in comparison to sections not exposed to immunogenic peptide, pre-incubation with antigenic peptide greatly reduced the labeling intensity of antibodies against ClC4–7. An assessment of ClC3 antibody labeling block by ClC3 peptide was not done because of the very low labeling intensity of ClC3 antibody in the chicken retina (see Fig. 3B). It is important to note that in the case of ClC5, pre-incubation with antigenic peptide did not reduce antibody labeling of ganglion cells as much as in other retinal cell types (Fig. 2B right, arrowheads) suggesting that some of this labeling might be nonspecific. Interpretation of ClC5 antibody labeling results in ganglion cells must therefore be made with caution.

### CLC transporter expression in adult chicken retina

To determine the distribution of CLC transporters in the avian retina, we used the polyclonal antibodies described above to label frozen sections of chicken retina. Examination of ClC3–7 antibody labeling in the mature chicken retina revealed differences in expression patterns among internally expressed CLC transporters. The micrographs in Figure 3A–F were all obtained using the same acquisition settings and brightness adjustments were made identically, with the exception of ClC7 which was acquired at a lower PMT gain to avoid saturation of the fluorescent signal. Figure 3A shows a section of chicken retina labeled only with secondary antibody. Fluorescent signal was restricted to photoreceptor oil droplets and outer segments, which are well-characterized auto-fluorescent features in the chicken retina (Fig. 3A). These photoreceptor components are often lost during tissue preparation and sectioning; as such, their presence is variable in later figures.

### ClC3

The polyclonal antibody raised against ClC3 c-terminus gave minimal labeling in vertical sections of chicken retinal tissue. Comparison to a control section obtained with the same acquisition settings (secondary antibody only, Figure 3A) showed that ClC3 antibody labeling was barely above background levels and was mainly restricted to the nuclear layers of the retina, where cell bodies reside (Fig. 3B, H, and N). A monoclonal antibody specific to a region of rat ClC3 n-terminus (identical to the chicken sequence) also minimally labeled chicken retina (Fig. S1) further supporting a low level of ClC3 expression in chicken retina.

### ClC4

A vertical section of retinal tissue labeled with ClC4 antibody showed that ClC4 was widely expressed in the nuclear and plexiform (synaptic) layers of the retina (Fig. 3C). Most immunoreactivity in the photoreceptors was limited to the outer nuclear layer (ONL), and labeling in the inner nuclear layer (INL) was stronger in the inner half of the INL (Figure 3C). Most cells in the ganglion cell layer (GCL) were ClC4 positive. In the outer plexiform layer (OPL), where photoreceptors make synapses with bipolar cells and horizontal cells, ClC4 antibody label was diffuse (Fig. 3I). In the inner retina, some ClC4 positive processes could be seen emanating from the INL into the distal (furthest from GCL) inner plexiform layer (IPL, Fig. 3O, arrows). In addition, there were two broad bands of ClC4 labeling in the IPL at 20–35% and 55–75% IPL depth as well as a thinner third band at approximately 95% IPL depth (Fig. 3O, 1–3, see Methods). Finally, very faint ClC4 antibody labeling was detected in the nerve fiber layer (NFL), which is primarily composed of ganglion cell axons (Fig. 3C).

### ClC5

**Figure 4:**
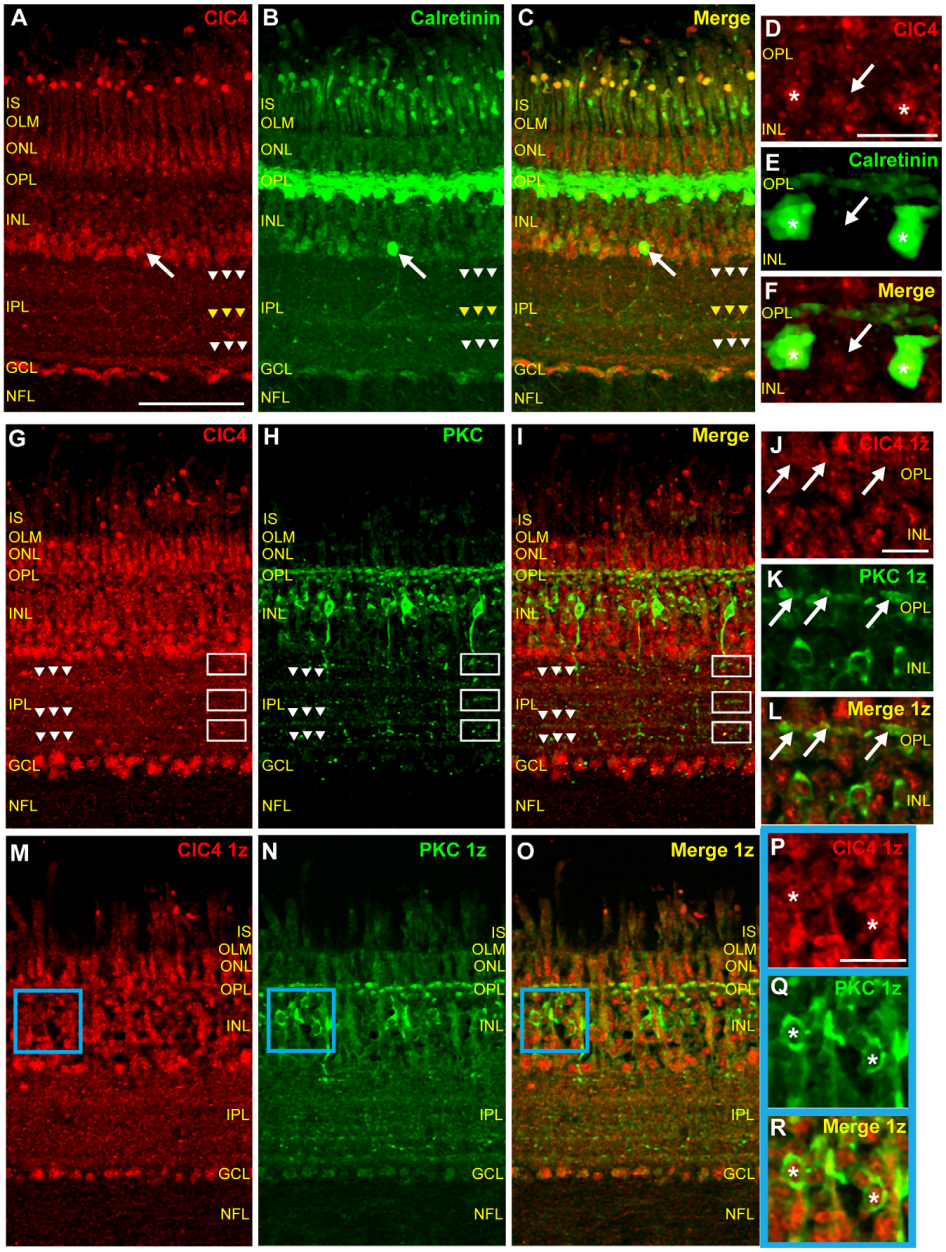
ClC4 is expressed in calretinin-positive horizontal cells and PKC-positive bipolar cells. **A–C**, Vertical section of mature chicken retina double-labeled with antibodies specific for ClC4 and calretinin. ClC4 antibody labeling co-localizes with anti-calretinin labeling in horizontal cells and amacrine cells (arrow) and cells in the GCL. White arrowheads indicate IPL bands immunoreactive for both antibodies. Yellow arrowheads indicate an IPL band immunoreactive only for the calretinin antibody. **D–F**, Lower gain, higher magnification images of horizontal cell labeling by ClC4 and calretinin antibodies show that ClC4 is expressed in both calretinin-positive (asterisks) and calretinin-negative (arrow) horizontal cells. **G–I**, Chicken retinal tissue labeled with the ClC4 antibody and with an antibody specific for PKC reveal overlapping ClC4 and PKC expression. Arrowheads indicate that PKC-positive bands in the IPL overlap with ClC4 IPL immunoreactivity. Boxes indicate ClC4-negative, PKC-positive bipolar cell terminals. **J–L**, Higher magnification, single z plane images of a different PKC and ClC4 antibody labeled section. Co-localization of ClC4 and PKC antibody in the OPL supports expression of ClC4 in bipolar cell dendrites (arrows). **M–O**, Images from a single z plane from the same z stack as in G–I. **P–R**, Zoomed in images of the blue-bordered area in M–O confirm that ClC4 is expressed in PKC-positive bipolar cell bodies (asterisks) in the chicken retina. (Scale in A–C, G–I, and M–O, 50 µm; in D–F, J–L, and P–R, 10 µm.)

Of all of the CLC antibodies examined in the adult chicken retina, the ClC5 had the broadest expression pattern. Similar to ClC4, the ClC5 antibody labeled tissue in all of the synaptic and nuclear layers of the adult chicken retina (Fig. 3D). However, the intensity of the labeling was much greater than that of ClC4. ClC5 expression was widespread in both photoreceptor inner segments and the outer nuclear layer, and there was particularly intense labeling of the outer limiting membrane (OLM, Fig. 3D, arrow). In the INL, the ClC5 labeling pattern was similar to that of ClC4, with more antibody signal in that layer concentrated in the inner half of the INL. As with anti-ClC4, ClC5 antibody labeled most cells in the GCL. ClC5 was expressed in the synaptic layers of the retina. ClC5 labeling was diffusely distributed throughout the OPL and was also found in scattered processes (Fig. 3J, arrowheads). In the inner retina, some isolated, thin ClC5-positive processes were observed extending vertically from the INL into varying depths of the IPL (Fig. 3P, arrows). ClC5 antibody also labeled processes in the distal IPL in a broad band extending from 0–25% IPL depth (Fig. 3P, 1) as well as three thin bands located at approximately 50% (Fig. 3P, 2), 60% (Fig. 3P, 3), and >90% (Fig. 3P, 4) IPL depths, with a fifth band at 70% IPL depth discernable in some sections (Fig. 3P, arrowhead). Finally, many process in the NFL were intensely labeled by the ClC5 antibody (Fig. 3D).

### ClC6

Like ClC3, the labeling intensity of the ClC6 antibody in the retina was much weaker than that of ClC4 and ClC5, though expression was faintly discernable in all of the nuclear and plexiform layers (Fig. 3E). Interestingly, however, ClC6 antibody labeling was more intense in photoreceptors and in puncta in the OPL (Fig. 3K, arrows). Furthermore, the photoreceptor labeling was restricted to vertical elements distributed diffusely in the proximal portion of photoreceptor inner segments and the distal portion of the ONL (Fig. 3E, arrowheads). This labeling was very different in appearance from the labeling of the OLM by the ClC5 antibody (Fig. 3D). Though the labeling intensity of the ClC6 antibody was weak in the IPL, three faint bands of immunoreactivity could be observed at approximately 20%, 65%, and >90% IPL depth (Fig. 3Q, 1–3). Panel brightness was enhanced to bring out ClC6 IPL expression (Fig. 3M and N showing no primary and ClC3 labeling were adjusted the same way). ClC6 antibody labeling was not observed in the NFL.

### ClC7

ClC7 antibody labeling was strong in most layers of the retina, but the labeling pattern was more punctate than that of the other CLCs (Fig. 3F). In photoreceptors, ClC7 labeling was distributed in bright puncta in photoreceptor inner segments as well as in vertical elements (Fig. 3F, arrowheads) around the inner segment/ONL border. However, ClC7 signal was very weak in the rest on the ONL (Fig. 3F). ClC7 immunoreactivity was strong in the outer and inner INL, but there was a band in roughly the center of the layer nearly devoid of labeling (Fig. 3F, white arrow). Bright punctate labeling was more or less evenly distributed in the outer INL whereas in the inner INL, ClC7 signal appeared to be particularly concentrated in some triangular cell bodies at the INL/IPL border (Fig. 3F, asterisks). Some of these triangular cells had puncta of ClC7 labeling in thick processes projecting into the IPL (Fig. 3F, yellow arrows). ClC7 labeling was observed in most cell bodies of the ganglion cell layer. ClC7 expression was diffusely distributed in the outer plexiform layer (Fig. 3L). In the IPL, there was a band of ClC7 immunoreactivity at approximately 15% depth (Fig. 3R, 1) and a thicker band between 30% and 90% depth (Fig. 3R, 2). Visible in some sections, including the one shown in Figure 3, was a narrow strip within this thicker band lacking ClC7 antibody label at approximately 70% IPL depth (Fig. 3R, 3). Interestingly, no ClC7-positive band was ever observed near the GCL (>90% IPL depth), a feature shared by ClC4–6. Scattered processes in the NFL were faintly ClC7-positive (Fig. 3F).

To determine the retinal cell types expressing different CLC transporters, we double labeled retinal tissue with the CLC transporter antibodies and antibodies raised against cell-type specific markers. Due to the low level of ClC3 antibody labeling in the chicken retina (see Fig. 3), we focused on characterizing the expression patterns of ClC4–7.

### Cell-type specific markers for chicken retina

From the literature, we identified a suite of marker proteins with established cell-type specific expression in chicken retina. Exposing retinal sections to monoclonal antibodies raised against these proteins gave results in good agreement with what has been previously reported by other groups using in the chicken retina. Below, we describe the labeling patterns of these antibodies and the cell-type specific expression of their target proteins in the chicken retina.

Calretinin is a calcium-binding protein expressed in a majority of horizontal cells as well as in subsets of bipolar cells, amacrine cells, and ganglion cells in the chicken retina [32], [33]. We used a monoclonal calretinin antibody raised against recombinant rat calretinin (>80% identity with chicken calretinin protein) to label calretinin expressing cells in vertical sections of chicken retinal tissue. Here, we used a monoclonal calretinin antibody to identify horizontal cells.

A monoclonal antibody specific for the alpha and beta isoforms of protein kinase C (PKC) has been shown to label two populations of chicken bipolar cells [34], [35], and our results with this antibody were similar to descriptions of PKC expression in the retina in these studies and in one using a different PKC antibody [36].

The HPC-1 monoclonal antibody binds syntaxin 1, a protein known to be expressed in amacrine cells and not in ganglion cells or bipolar cells [37], [38]. This antibody has been extensively used as a specific marker for amacrine cells in a number of different species, including the chicken [39].

Glutamine synthetase (GS) converts ammonia and glutamate into glutamine and is a useful glial cell marker in the central nervous system. In the chicken retina, GS is expressed by Müller glial cells [40], [41] and possibly by another population of astrocyte-like glial cells in the GCL and NFL [42]. We used a monoclonal antibody specific for glutamine synthetase to selectively label Müller cells in the chicken retina.

Our interest in understanding the role of CLCs in the NO-dependent release of Cl^−^ from internal stores motivated us to examine the relative distribution of CLC transporters and the neuronal isoform of nitric oxide synthase (nNOS), an enzyme responsible for catalyzing the synthesis of NO in the presence of calcium and calmodulin in the CNS. In a previous investigation of nNOS expression in chicken retina, Fischer et al [43] described four distinct types of nNOS positive amacrine cells using two different polyclonal nNOS antibodies. They also observed nNOS expression in efferent target cells (ETCs), in several bands of the IPL, in the OPL, and in a subset of ganglion cells and photoreceptors. Using a different polyclonal nNOS antibody in a later study, we found a similar expression pattern [44]. Here, we used a monoclonal nNOS antibody that strongly co-localized with the polyclonal nNOS antibody from the 2003 study (data not shown) to label nNOS-expressing cells in the retina.

SV2 is expressed in photoreceptor terminals, but not in horizontal cells in the chicken retina [45] and a monoclonal SV2 antibody has been used as a marker for photoreceptor terminals in chicken retina [46], [47]. In the current work, we used the monoclonal antibody specific for SV2 to examine the localization of ClC6 expression in the OPL.

The labeling pattern of a polyclonal choline acetyltransferase (ChAT)-specific antibody has been well characterized in the chicken retina [25], [48]. Here, we used the same ChAT antibody to examine the expression of CLC transporters in cholinergic amacrine cells.

### ClC4 is expressed in calretinin-positive horizontal cells

Figures 4B and E show that horizontal cell labeling with the monoclonal calretinin antibody was in good agreement with the findings of Fischer et al [33] and Ellis et al [32]. In Figure 4E, a high magnification image, acquired at a lower gain than the image shown in 4B, shows intensely-labeled, calretinin-positive horizontal cells as well as diffuse immunoreactivity in 2–3 strata of the OPL. Labeling of putative bipolar cells, amacrine cells, and ganglion cells was also similar to that found in these previous investigations, but certain elements in the IPL were less well-resolved, perhaps due to the monoclonal nature of the antibody. ClC4 antibody labeling co-localized with the calretinin signal in horizontal cells (Fig. 4D–F, asterisks) as well as in strongly calretinin-positive cell bodies near the INL/IPL border (Fig. 4A–C, white arrow) and in more weakly immunoreactive cells in the inner half of the INL and in the GCL. Note that ClC4 was expressed in calretinin-negative horizontal cells as well (Fig. 4D–F, white arrow). The calretinin antibody also labeled processes projecting from the INL to a narrow band of immunoreactivity centered on 55% IPL depth (Fig. 4B, yellow arrowheads) as well as two much fainter, broad bands in the IPL, one centered around 25% and one centered around 90% IPL depth (Fig. 4B, white arrowheads). There was some overlap of ClC4 and calretinin expression in the distal and proximal IPL, but not, interestingly, in the middle band (55% IPL depth) of calretinin immunoreactivity, which, in Figure 4B and C is the most clearly resolved calretinin-positive IPL band (yellow arrowheads). Co-localization of ClC4 and calretinin in the OPL was minimal (Fig. 4D–F).

### ClC4 is expressed in PKC-positive bipolar cells

To determine whether ClC4 is expressed in bipolar cells of the chicken retina, we double labeled retinal sections with ClC4 antibody and PKC antibody. Figures 4H (projection image of a stack of twenty z planes through the retinal cross-section) and 4N and Q (images of a single z plane from the same stack) show that the PKC antibody strongly labeled a small number of cell bodies in the middle of the INL with projections to the OPL and IPL and more faintly labeled a larger number of cell bodies in the distal half of the INL (Fig. 4H). A few scattered cell bodies close to the INL/IPL border were also faintly PKC positive, possibly representing PKC-expressing amacrine cells (Fig. 4H, [36]). In the OPL, two bands of PKC immunoreactivity were observed (Fig. 4K, high magnification image of a single plane from a different retinal section). In the IPL, there were three distinct layers of PKC positive bipolar cell terminals at approximately 0–10%, 45%, and 80% IPL depth (ovals, Fig. 4H) as well as three much weaker bands of immunoreactivity at 30%, 70%, and 92% IPL depth (arrowheads, Fig. 4H). Figures 4P–R show that the brightly labeled, PKC positive cell bodies in the INL were also labeled with ClC4 antibody (asterisks). ClC4 antibody signal was typically surrounded by PKC antibody signal in these cells. PKC and ClC4 displayed some overlapping expression in the OPL although the ClC4 antibody labeling there was more diffuse than the PKC labeling (Fig. 4J–L, white arrows). In the IPL, ClC4 did not co-localize strongly with PKC in bipolar cell terminals (Fig. 4G–I, boxes). However, the two antibodies did appear to overlap in the more weakly PKC-positive IPL bands (Fig. 4G–I, arrowheads). Taken together, these results suggest that ClC4 is expressed in at least two populations of bipolar cells in the chicken retina as well as in PKC-positive bands in the IPL.

### ClC4 is expressed in retinal amacrine cells

**Figure 5:**
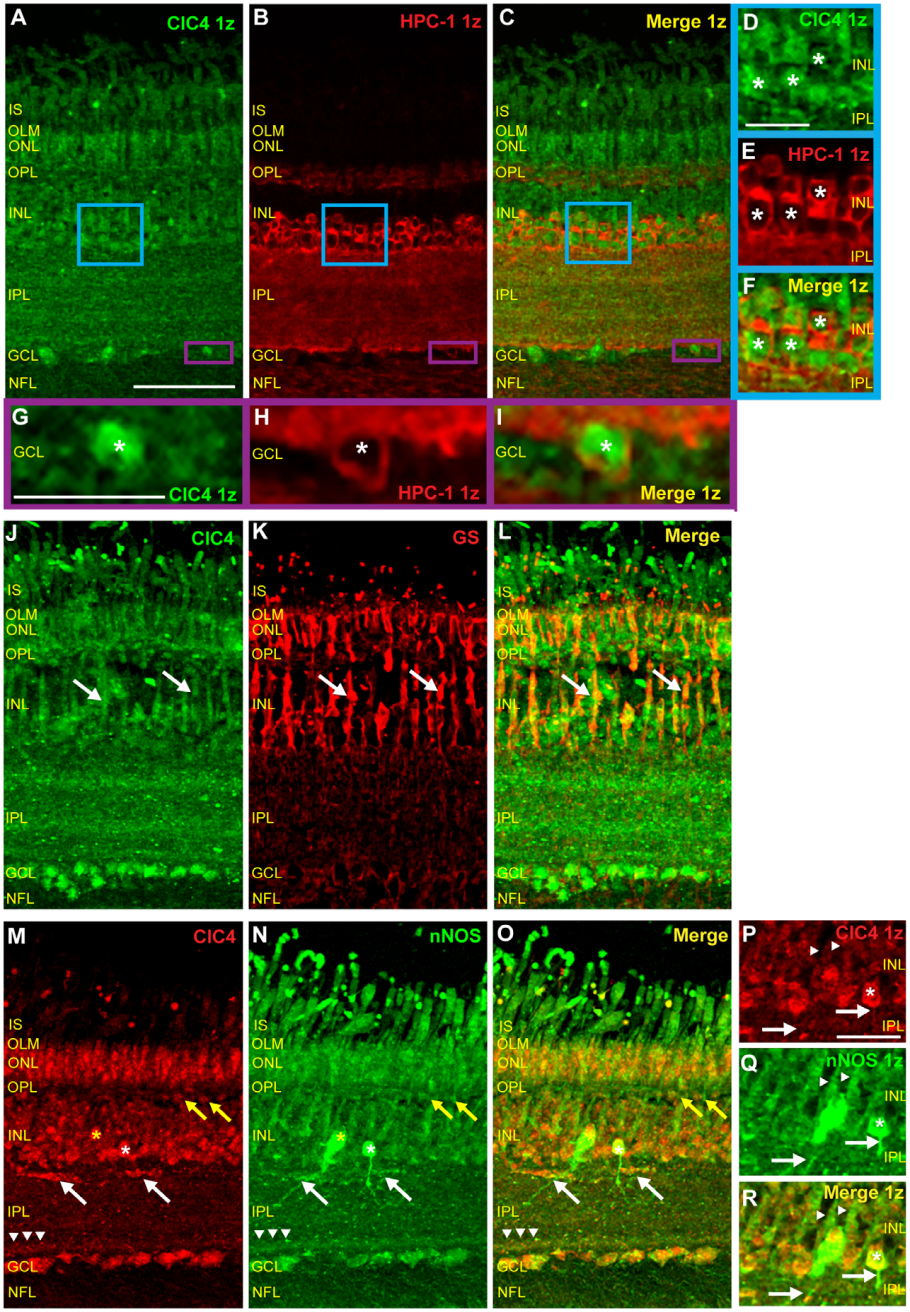
ClC4 is expressed in amacrine cells, Müller glia, and nNOS-positive cells. **A–C**, Single z plane images of chicken retinal tissue sections double-labeled with antibodies specific for ClC4 and the amacrine cell marker, HPC-1 reveal that ClC4 is expressed in the HPC-1-positive cells. **D–F**, Zoom images of ClC4 and HPC-1 antibody labeling of the blue box region in A–C. ClC4 is expressed in amacrine cells, predominantly at cytosolic locations (asterisks). **G–I**, Zoomed images of the purple box region in A–C show that ClC4 is expressed in displaced amacrine cells in the retina (asterisk). **J–L**, Retinal tissue double-labeled with ClC4 antibody and an antibody specific for the Müller cell marker, glutamine synthetase (GS). Arrows indicate examples of ClC4 expression in Müller glia. **M–O**, Retinal sections double-labeled with ClC4 antibody and a nNOS-specific antibody. The nNOS antibody labels cells in all layers of the chicken retina, with strong labeling of horizontal cells (yellow arrows), a subset of amacrine cells and processes (white asterisk) as well as putative Müller cells, efferent target cells (ETCs, yellow asterisk), IPL processes (white arrows) and bands, ganglion cells, and scattered processes in the NFL. ClC4 is expressed in almost all nNOS-expressing cells. Note co-localization of ClC4 and nNOS in an immunoreactive band near to the IPL/GCL boundary (arrowheads). **P–R**, Single z plane zooms of ClC4 and nNOS antibody labeling confirms co-labeling of glial cells (arrowheads) and IPL processes (white arrows). (Scale in A, 50 µm, applies to B–C, J–L, and M–O; Scale D–F, 15 µm; Scale G–I, 15 µm; Scale P–R, 15 µm.)

The labeling of the proximal half of the INL (closest to the IPL) by the ClC4 antibody (Fig. 3C) suggests that this protein may be expressed in retinal amacrine cells. To examine the expression of ClC4 in amacrine cells, we double-labeled chicken retinal sections with ClC4 antibody and the antibody specific for syntaxin 1 (HPC-1). Figures 5B and 5E (asterisks) show HPC-1 antibody labeling in the cell membranes of cell bodies in the inner half of the INL. There was also extensive labeling throughout the IPL as well as fainter labeling in the OPL in putative horizontal cell terminals [49]. Scattered cell bodies in the GCL, representing displaced amacrine cells, were also labeled (Fig. 5H, asterisk). All HPC-1 expressing cells in the INL and GCL also appeared to express ClC4, confirming that ClC4 is expressed in retinal amacrine cells (Fig. 5C, F, and I).

### ClC4 is expressed in glial cells in the chicken retina

Figures 5J–L show a retinal tissue section double labeled with ClC4 antibody and the monoclonal antibody specific for glutamine synthetase (GS). In Figure 5K, a projection image of a retinal section labeled with a GS-specific monoclonal antibody showed the expected pattern of labeling in Müller cells [41], with GS expression in processes stretching from the OLM to the inner limiting membrane (ILM, not shown) that thickened in putative perikarya in the center of the INL. ClC4 and GS antibody signals co-localized in some areas of the INL and outer retina, specifically in Müller cell perikarya (Fig. 5J–L, white arrows) and in glial processes in the OPL and ONL, indicating that ClC4 is expressed in Müller cells of the chicken retina.

### ClC4 transporters are present in nNOS-expressing cells in the retina

To examine ClC4 expression in retinal cells containing nNOS, we double-labeled retinal tissue with monoclonal nNOS antibody and ClC4 antibody (Fig. 5M–R). ClC4 antibody signal co-localized with that of nNOS in the ONL, putative horizontal cells (Fig. 5M–O, yellow arrows), Müller cells (Fig. 5P–R, arrowheads), some amacrine cell bodies and processes at the INL/IPL border (Fig. 5O and R, white asterisk and arrows) and putative efferent target amacrine cells (ETCs, [43], Fig. 5O, yellow asterisk). ClC4 and nNOS expression also overlapped in the ClC4 positive band closest to the GCL (Fig. 5M–O, arrowheads). ClC4 and nNOS were also both expressed in the GCL. Taken together, these results support the possibility of interaction between ClC4 proteins and NO produced in nNOS-expressing cells in the chicken retina.

### ClC5 is expressed in calretinin-positive horizontal cells

**Figure 6:**
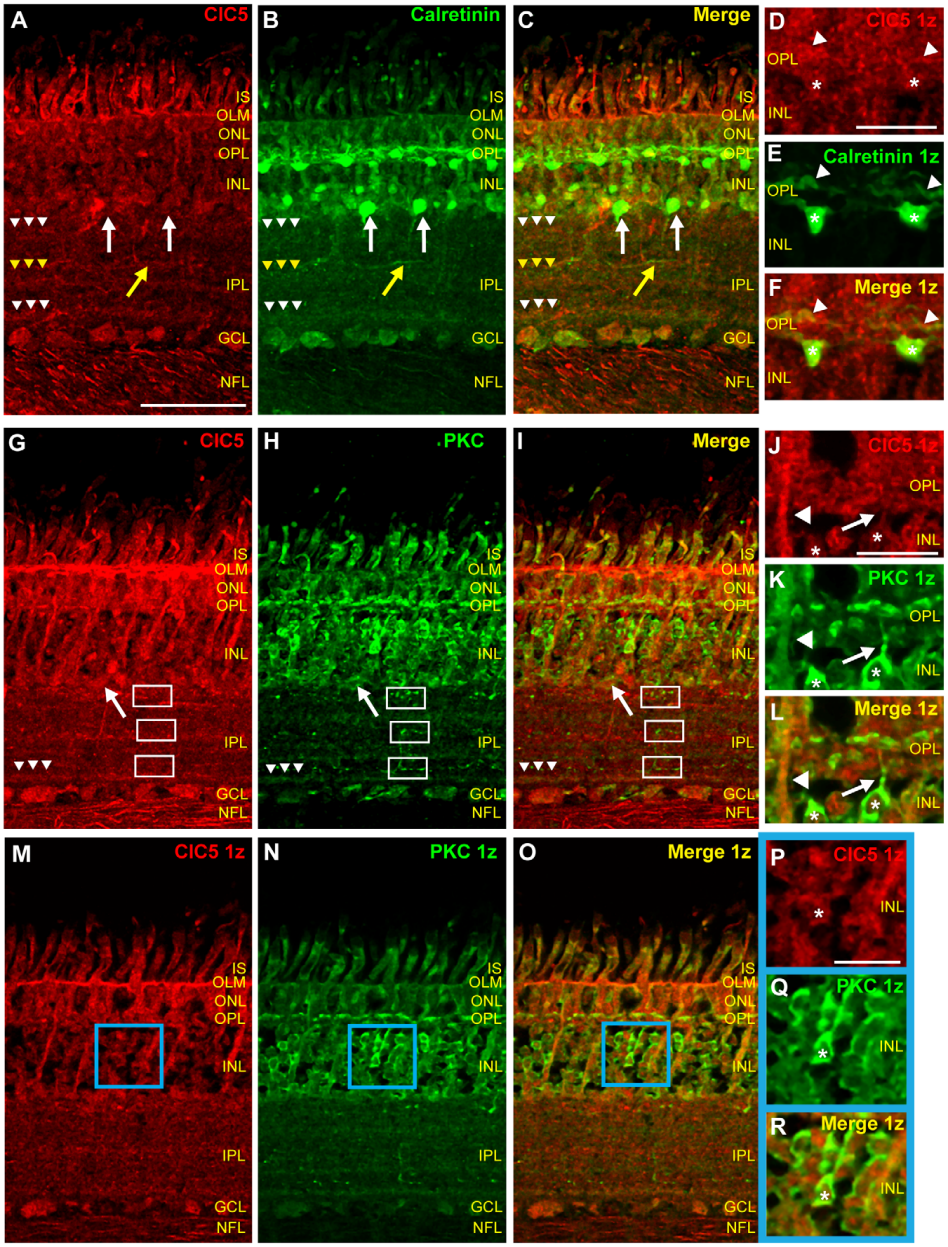
ClC5 is expressed in calretinin-positive horizontal cells and PKC-positive bipolar cells. **A–C**, Retinal tissue sections double-labeled with ClC5 and calretinin antibodies. ClC5 antibody labeling co-localizes with that of calretinin in horizontal cells and amacrine cells (cell body, white arrow; process, yellow arrow). White arrowheads indicate IPL bands immunoreactive for both antibodies. Yellow arrowheads indicate an IPL band that is only immunoreactive for the calretinin antibody. **D–F**, Lower gain, higher magnification images from a different double-labeled tissue section show that ClC5 is expressed in calretinin-positive horizontal cell bodies (asterisks) and processes (arrowheads). **G–I**, Retinal sections labeled with the ClC5 antibody and an antibody specific for PKC reveal overlapping ClC5 and PKC expression. Arrowheads indicate that PKC-positive bands in the IPL overlap with ClC5 IPL immunoreactivity only in the band nearest the GCL. White arrow indicates putative PKC-positive amacrine cell. Boxes indicate ClC5-negative, PKC-positive bipolar cell terminals. **J–L**, Higher magnification, single z plane images of ClC5 and PKC antibody labeling in the OPL and outer INL in a different tissue section. ClC5 and PKC antibody signals are co-localized in bipolar cell bodies (asterisks) and OPL projections (arrow) as well as in glial projections (arrowhead). **M–O**, Images from a single z plane from the same z stack as in G–I. **P–R**, Zoom in of the blue-bordered area in M–O confirms that ClC5 is expressed in PKC-positive bipolar cell bodies (asterisk) in the chicken retina. (Scale A–C, 50 µm, applies for G–I and M–O as well; Scale D–F, 10 µm; Scale J–L, 15 µm; Scale P–R, 10 µm.)

To begin to examine ClC5 expression in the outer retina, we double-labeled retinal tissue with ClC5 and calretinin antibodies. All calretinin-positive horizontal cell bodies were also ClC5-positive, and processes in the OPL were labeled by both antibodies, confirming that ClC5 was expressed in most horizontal cells in the chicken retina (Fig. 6A–F). In addition, images of a retinal cross section labeled with the ClC5 and calretinin antibodies (Fig. 6A–C) demonstrated co localization of ClC5 and calretinin in cell bodies in the inner half of the INL and in the GCL. INL cell bodies brightly labeled with calretinin antibody were also faintly labeled with ClC5 antibody (Fig. 6A–C, white arrows). Calretinin and ClC5 antibodies also overlapped in putative glial cell processes and perikarya in the INL, OPL, and ONL (Fig. 6A–C). ClC5 antibody labeling co-localized with calretinin expression in the outer and inner calretinin-positive IPL bands (Fig. 6A–C, white arrowheads), but not as much in the central IPL band (Fig. 6A–C, yellow arrowheads). However, some individual neuronal processes projecting to the middle of the IPL were labeled with both antibodies (Fig. 6A–C, yellow arrows). Finally, we observed co-labeling of the two antibodies in many ganglion cell axons as well.

### ClC5 is present in PKC-expressing bipolar cells

Consistent with ClC5 expression in chicken bipolar cells, double labeling of retinal sections with ClC5 and PKC antibodies revealed overlapping expression of the two proteins in bipolar cell bodies in the outer half of the INL (Fig. 6L and R, asterisks). PKC-positive cell bodies in the inner INL were also labeled with ClC5 antibody, suggesting that amacrine cells that express PKC also express ClC5 (Fig. 6G–I, white arrow). In single plane images of the OPL (Fig. 6J–L), the ClC5 antibody signal co-localized with that of PKC in bipolar cell dendrites. ClC5 and PKC antibody labeling also overlapped in Müller cell processes projecting to the OLM (Fig. 6J–L, arrowhead). In the IPL, in contrast to our ClC4/PKC co-labeling observations, overlapping ClC5 and PKC antibody labeling was only observed in the immunoreactive band nearest to the GCL (arrowheads). The other PKC- and ClC5-positive bands were intercalated in the IPL, and ClC5 was not present in bipolar cell terminals (Fig. 6G–I, boxes).

### ClC5 transporters are expressed in retinal amacrine cells

**Figure 7:**
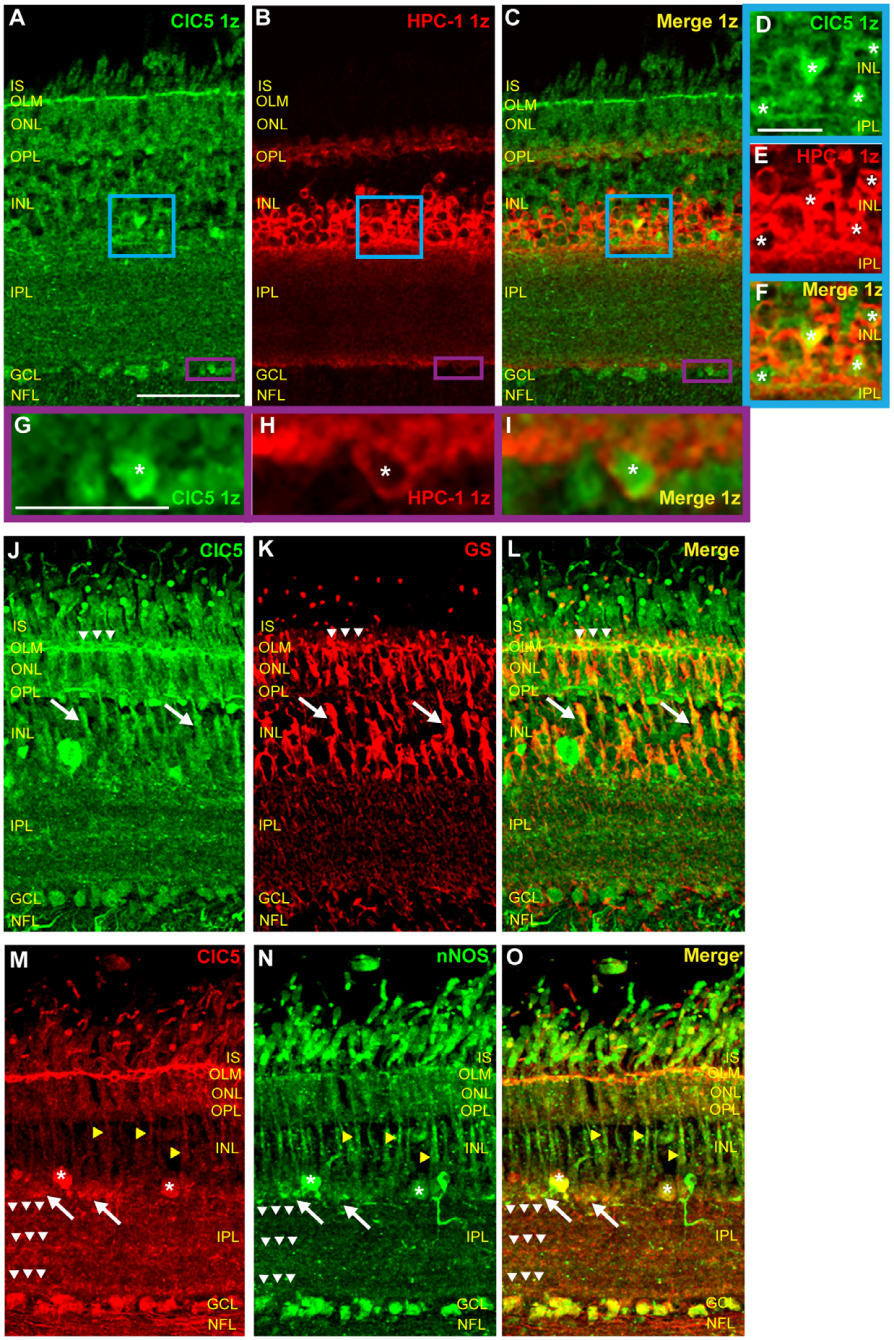
ClC5 is expressed in amacrine cells, Müller glia, and nNOS-positive cells. **A–C**, Single z-plane images of chicken retinal tissue sections double-labeled with ClC5 and HPC-1 antibodies reveal that ClC5 is expressed in the HPC-1-positive cells. **D–F**, Zoom of ClC5 antibody labeling of blue box region in A–C shows that ClC5 is expressed cytosolically in amacrine cells (asterisks). **G–I**, Zoom of purple box region in A–C shows ClC5 antibody labeling in the ganglion cell layer, indicating that ClC5 is expressed in displaced amacrine cells in the retina (asterisk) as well as in neighboring, HPC-1 negative ganglion cells. **J–L**, Sections of retinal tissue double-labeled with ClC5 and GS antibodies show that GS and ClC5 co localize in Müller cells (arrows) and in the OLM (arrowheads). **M–O**, Retinal sections double-labeled with ClC5 and nNOS antibodies reveal expression of ClC5 in nNOS-expressing cells. Yellow arrowheads and asterisks indicate overlapping signal in Müller cells and amacrine cells, respectively. Arrows indicate co-expression in puncta near the INL/IPL border, and white arrowheads indicate co-localization in three bands in the IPL. (Scale A–C, 50 µm, applies to J–L and M–O as well; Scale D–F, 15 µm; Scale G–I, 15 µm.)

In order to confirm ClC5 expression in retinal amacrine cells, we used the HPC-1 antibody to selectively label amacrine cells. Figures 7A–I show single z plane images of tissue sections doubled-labeled with HPC-1 antibody and ClC5 antibody. HPC-1 signal enveloped ClC5 signal in cell bodies in the INL and GCL, indicating that, as with ClC4, ClC5 was expressed by amacrine cells in the INL and displaced amacrine cells in the GCL.

### ClC5 transporters are expressed in Müller glia

To determine whether ClC5 is expressed in glial cells, glutamine synthetase antibody was used to selectively label Müller cells in chicken retinal cross sections (Fig. 7J–L). In this section, ClC5 antibody labeling co-localized with that of glutamine synthetase, indicating that ClC5 is expressed in Müller cells (Fig. 7J–L, white arrows). ClC5 and glutamine synthetase antibody labeling also strongly overlapped in the OLM (Fig. 7J–L, arrowheads).

### nNOS expressing cells also express ClC5

We have demonstrated widespread overlapping ClC4 and nNOS expression in the adult chicken retina. Is ClC5 localized to nNOS-expressing cells in the retina as well? Figures 7M–O show ClC5 antibody labeling in nNOS-positive amacrine cells (asterisks), in Müller cells (yellow arrowheads), in scattered processes and puncta near the INL/IPL border (arrows), and in ganglion cells. In the IPL, CLC5 immunoreactive bands were also immunoreactive for nNOS (white arrowheads). Taken together, these results support the possibility of interaction between ClC5 transporters and NO produced in nNOS-expressing cells.

### ClC6 is expressed in photoreceptor terminals

**Figure 8:**
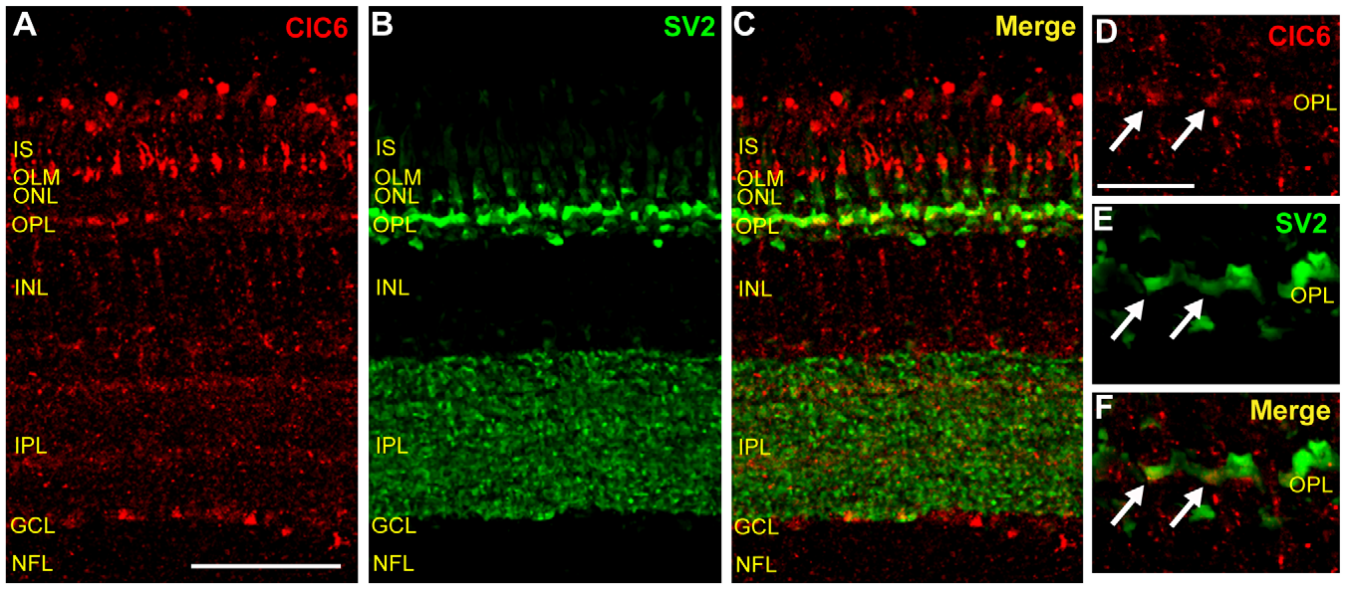
ClC6 is expressed in photoreceptor terminals in the OPL. **A–C**, A vertical section of adult chicken retina double-labeled with ClC6 and a monoclonal antibody specific for the synaptic vesicle protein, SV2 (Scale, 50 µm). Note ClC6-positive vertical elements near the OLM as well as ClC6-positive puncta in the OPL. **D–F**, Higher magnification images of the OPL. Arrows indicate expression of ClC6 in photoreceptor terminals. (Scale D–F, 15 µm).

In comparison to other CLC transporters, ClC3 and ClC6 did not appear to be widely expressed in the avian retina (Fig. 3). ClC3 antibody labeling was uniformly weak in all regions of the chicken retina. However, ClC6 antibody labeling, though low in most parts of the retina, was intense in vertical elements in photoreceptors and somewhat more modestly present in the OPL. To further examine ClC6 expression in the OPL, retinal tissue sections were double labeled with ClC6 antibody and the monoclonal antibody to the synaptic vesicle protein SV2. Figure 8 shows SV2 antibody labeling in the OPL and IPL in cross-sections of retinal tissue. A higher magnification image revealed SV2 antibody labeling in the OPL is restricted to crescent-shaped elements consistent with photoreceptor terminals (Fig. 8E). Some of these SV2 positive elements overlapped with ClC6 antibody label (Fig. 8F, arrows), suggesting that ClC6 is expressed in photoreceptor terminals in the chicken retina.

### ClC7 is not expressed in calretinin-positive horizontal cells

**Figure 9:**
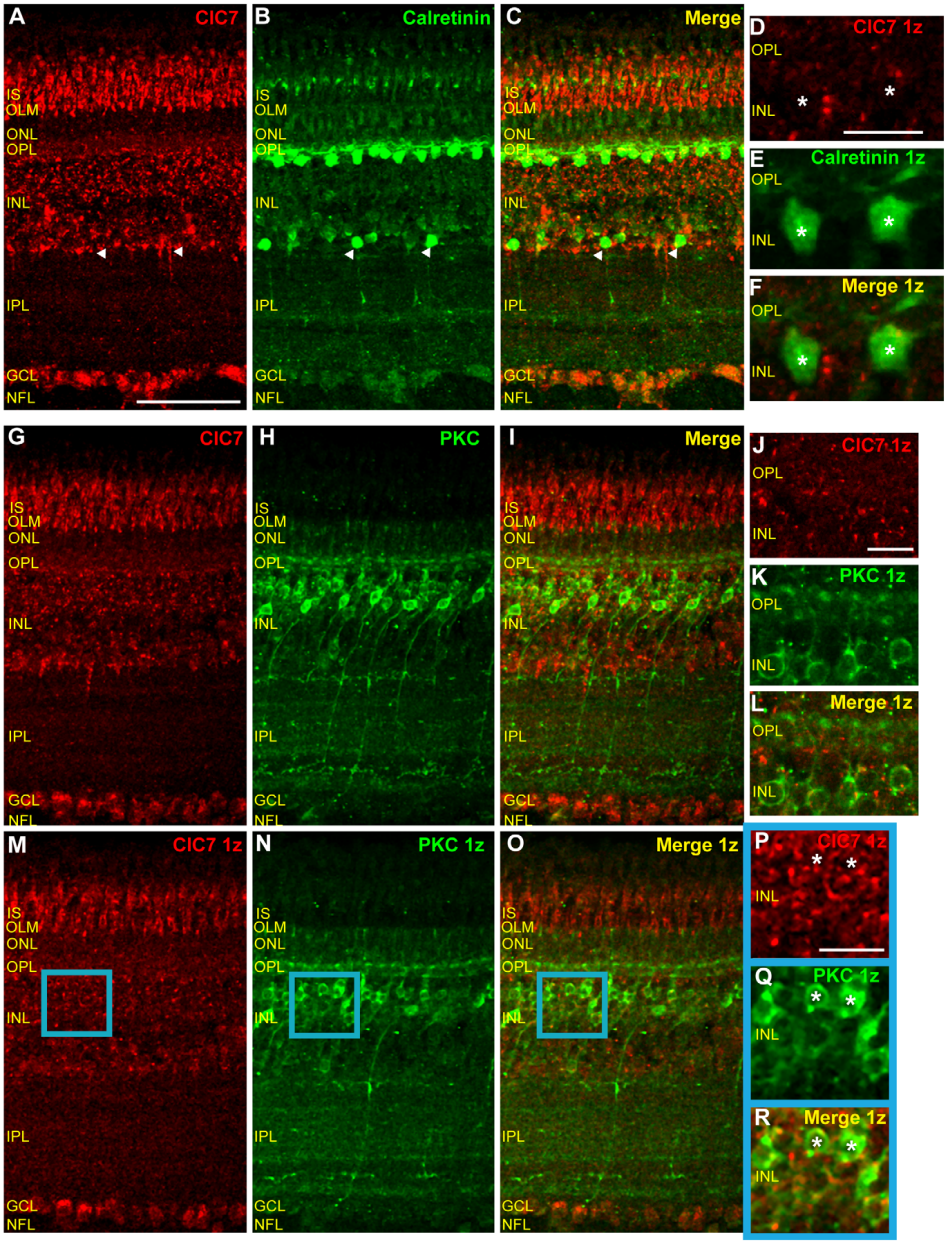
ClC7 is narrowly expressed in PKC-positive bipolar cells, and is absent from calretinin-positive horizontal cells. **A–C**, Retinal sections double-labeled with ClC7 and calretinin antibodies show that ClC7 antibody labeling is not strong in calretinin-positive cells. Arrowheads indicate strongly calretinin-positive amacrine cells that do not express ClC7. **D–F**, ClC7 is not expressed in calretinin-positive horizontal cells (asterisks). **G–I**, Retinal section labeled with ClC7 and PKC antibodies reveal minimal co-localization of the two antibodies. **J–L**, Higher magnification, single z plane images of a different tissue section show no co-expression of ClC7 and PKC in the OPL. **M–O**, Images from a single z plane from the same z stack as in G–I. **P–R**, Zoomed images of the blue-bordered area in M–O show little overlap in the signals of the two antibodies, although the asterisks indicate examples of ClC7 expression in the membrane of a PKC-positive cell. (Scale in A, 50 µm, applies to B–C, G–I, and M–O as well; Scale D–F, 15 µm; Scale J–L, 10 µm; Scale P–R, 15 µm.)

Images of tissue sections double-labeled with antibodies specific for calretinin and ClC7 show that, unlike ClC4 and ClC5, ClC7 is not expressed in calretinin-positive horizontal cells (Fig. 9D–F, asterisks). In addition, there was virtually no labeling of calretinin-expressing amacrine cells by the ClC7 antibody (Fig. 9A–C, arrowheads). However, calretinin-positive ganglion cells were also ClC7-positive. Finally, the labeling patterns of the two antibodies did not overlap appreciably in Müller cells or in the synaptic layers of the retina.

### ClC7 is expressed in PKC-positive bipolar cells at putative membrane locations

To further characterize ClC7 expression in the retina, we next examined ClC7 antibody labeling in bipolar cells labeled with the antibody specific for PKC. In Figures 9P–R, PKC-positive bipolar cells were faintly labeled by ClC7 antibody (asterisk), indicating that at least a subset of bipolar cells in chicken retina expresses ClC7. However, in these cells, ClC7 antibody label was confined to the margins of the cell body, perhaps in close association with the plasma membrane, an observation that differed from the internal cell body expression observed with ClC4 and ClC5. Furthermore, ClC7 antibody did not label PKC-expressing bipolar cell projections to the OPL or PKC-positive puncta in the OPL (Fig. 9J–L). In the inner retina, ClC7 expression did not overlap with PKC expression in the IPL (Fig. 9G–I), but ClC7-positive cells in the ganglion cell layer were also labeled by the PKC antibody.

### ClC7 is expressed in retinal amacrine cells

**Figure 10:**
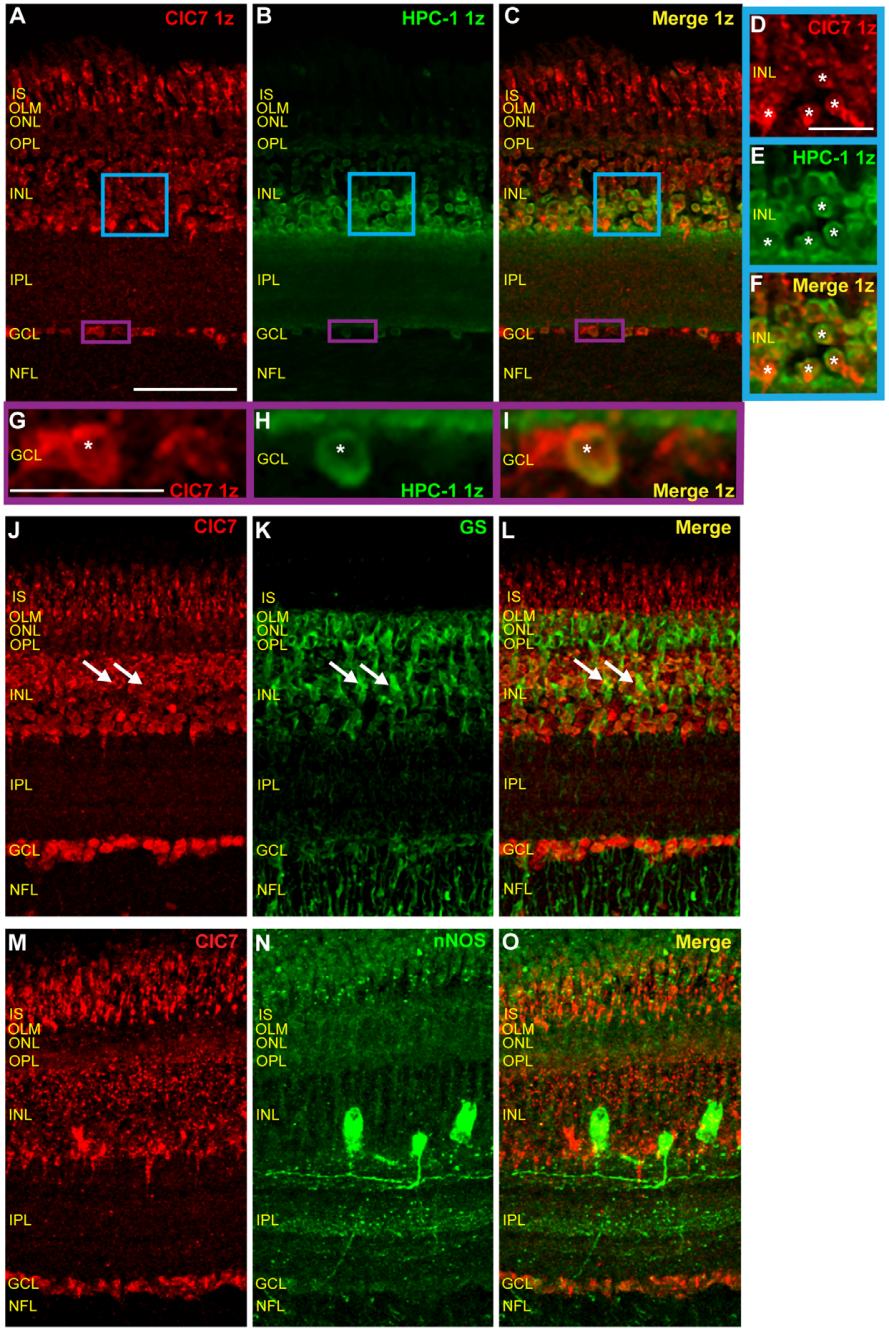
ClC7 is expressed in amacrine cells, but not widely expressed in Müller or nNOS-positive cells. **A–C**, Single z plane images of chicken retinal tissue sections double-labeled with ClC7 and HPC-1 antibodies show that ClC7 is expressed in HPC-1-positive cells. **D–F**, Zoom of blue box region in A–C shows that ClC7 is expressed in amacrine cells (asterisks), particularly those with cell bodies close to the IPL. **G–I**, Zoom of purple box region in A–C shows ClC7 antibody labeling in displaced amacrine cells of the ganglion cell layer (asterisk) as well as in neighboring, HPC-1 negative ganglion cells. **J–L**, Sections of retinal tissue double-labeled with ClC7 and GS antibodies. GS and ClC7 co-expression is limited to Müller cell margins in the center of the INL (arrows). **M–O**, Retinal sections double-labeled with ClC7 and nNOS antibodies (maximum projection images) reveal that, except for in the GCL, ClC7 is minimally expressed in nNOS-expressing cells. (Scale A–C, 50 µm, applies to J–L and M–O as well; Scale D–F, 15 µm; Scale G–I, 15 µm.)

Figures 10A–I show retinal tissue double-labeled with ClC7 antibody and with the amacrine cell marker antibody, HPC-1. Zoomed-in images of the INL (Fig. 10D–F) and the GCL (Fig. 10G–I) revealed ClC7 antibody labeling in amacrine cell bodies, confirming ClC7 expression in retinal amacrine cells.

### ClC7 is not widely expressed in retinal glial cells

Using an antibody specific for GS to selectively label glial cells, we observed some co-localization of ClC7 and GS antibodies in putative Müller cell perikarya in the adult chicken retina (Fig. 10J–L, arrows), but the ClC7 antibody labeling was very discrete and punctate and could have represented ClC7 expression in closely associated amacrine cells. Furthermore, ClC7 and glutamine synthetase antibody signals did not overlap in glial processes. Thus, ClC7 may be expressed in Müller glia in the chicken retina, but its expression pattern in these cells is much more restricted than that of ClC4 and ClC5.

### ClC7 is not widely expressed in nNOS-positive cells in the retina

To examine ClC7 transporter expression in nNOS-expressing cells, retinal tissue sections were double-labeled with antibodies to nNOS and ClC7. Figures 10M–O show that compared to ClC4 and ClC5, co-localization of ClC7 and nNOS was not very striking. This apparent difference was partially due to more restricted ClC7 expression in glial cells and partially due to the more discrete nature of the ClC7 signal. ClC7 antibody sparsely labeled nNOS-expressing photoreceptors, putative ETCs, and cells in the GCL (Fig. 10M–O). nNOS-positive processes and bands in the IPL were not labeled by ClC7 antibody. The triangle-shaped, strongly ClC7-positive cells at the INL/IPL border were not nNOS-positive.

### ClC5 and ClC7 are expressed in cholinergic cells in the retina

**Figure 11:**
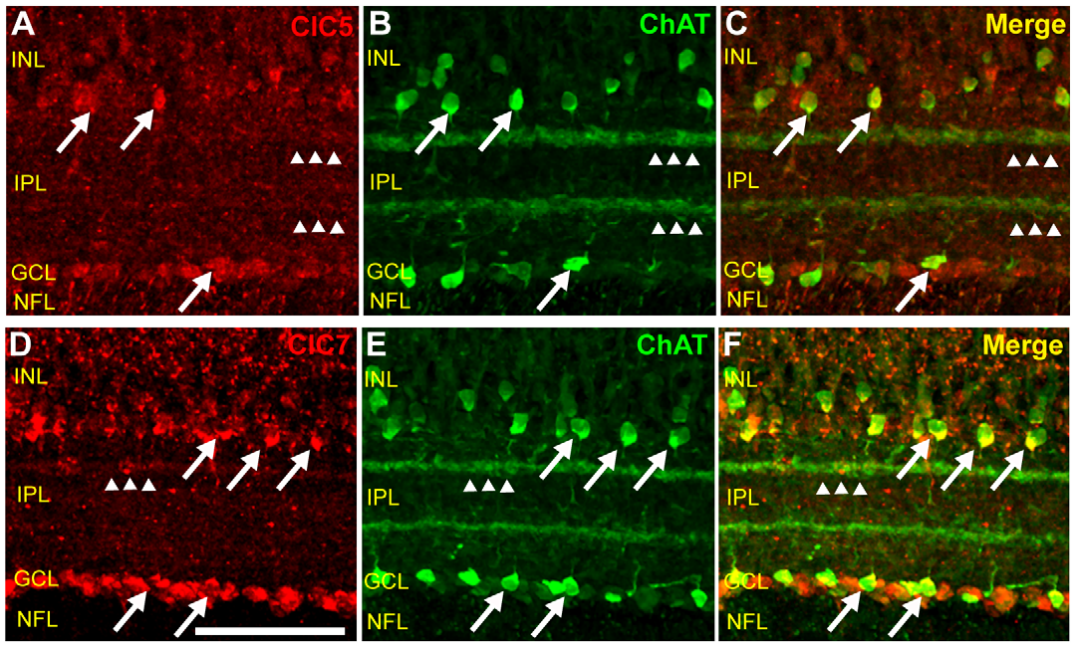
ClC5 and ClC7 are expressed in cholinergic cells of the chicken retina. **A–C**, Vertical section of chicken retinal tissue double-labeled with ClC5 antibody and a polyclonal antibody specific for ChAT. ChAT-positive amacrine cells express ClC5 (arrows). IPL bands immunoreactive for ChAT are also immunoreactive for ClC5 (arrowheads). **D–F**, Retinal tissue double-labeled with ClC7 and ChAT antibodies shows that ClC7 expression in the INL is highly co-localized with ChAT (arrows). ClC7 is also highly expressed in the GCL and in the outermost ChAT-immunoreactive IPL band (arrowheads). (Scale for A is 50 µm and applies for B–F as well.)

We have shown that both ClC5 and ClC7 were expressed in amacrine cells near the INL/IPL border and in displaced amacrine cells in the GCL (Fig. 7A–I, Fig. 10A–I). We have also shown that both antibodies labeled thin bands in the distal IPL (Fig. 3D and F). To further examine which types of amacrine cells express ClC5 and ClC7, we double-labeled chicken retinal tissue sections with antibodies to ChAT and the two CLC proteins. In our hands, the polyclonal ChAT antibody gave the expected labeling pattern in the inner retina, with two layers of widely spaced, brightly labeled cell bodies in the inner half of the INL and a layer of cells in the GCL sending projections out to IPL bands at approximately 20% and 65% depth, respectively (Fig. 11B and E). Because the ClC7 antibodies and the ChAT antibody were all raised in rabbit and because the only commercially available monoclonal ChAT antibody that we were able to find did not label bands in the IPL (Clone 1e6, data not shown), we performed sequential double labeling experiments in which the double labeled tissue was compared to side-by-side single-labeled controls. Figures 11A–C show that all ChAT-positive cells were also ClC5-positive (arrows) and that two of the ClC5 antibody-labeled bands in the IPL overlapped with the two ChAT-positive bands (arrowheads). These results are not surprising given that ClC5 seemed to be present to some degree in virtually all amacrine cells (See Figure 7A–I). Interestingly, however, ClC7 antibody labeling appeared to be particularly intense in ChAT-positive amacrine cells (arrows, Fig. 11D–F), suggesting that the protein might have a particular function in these cells. The thin outermost ClC7-positive IPL band was also labeled with ChAT antibody (arrowheads).

## Discussion

**Figure 12:**
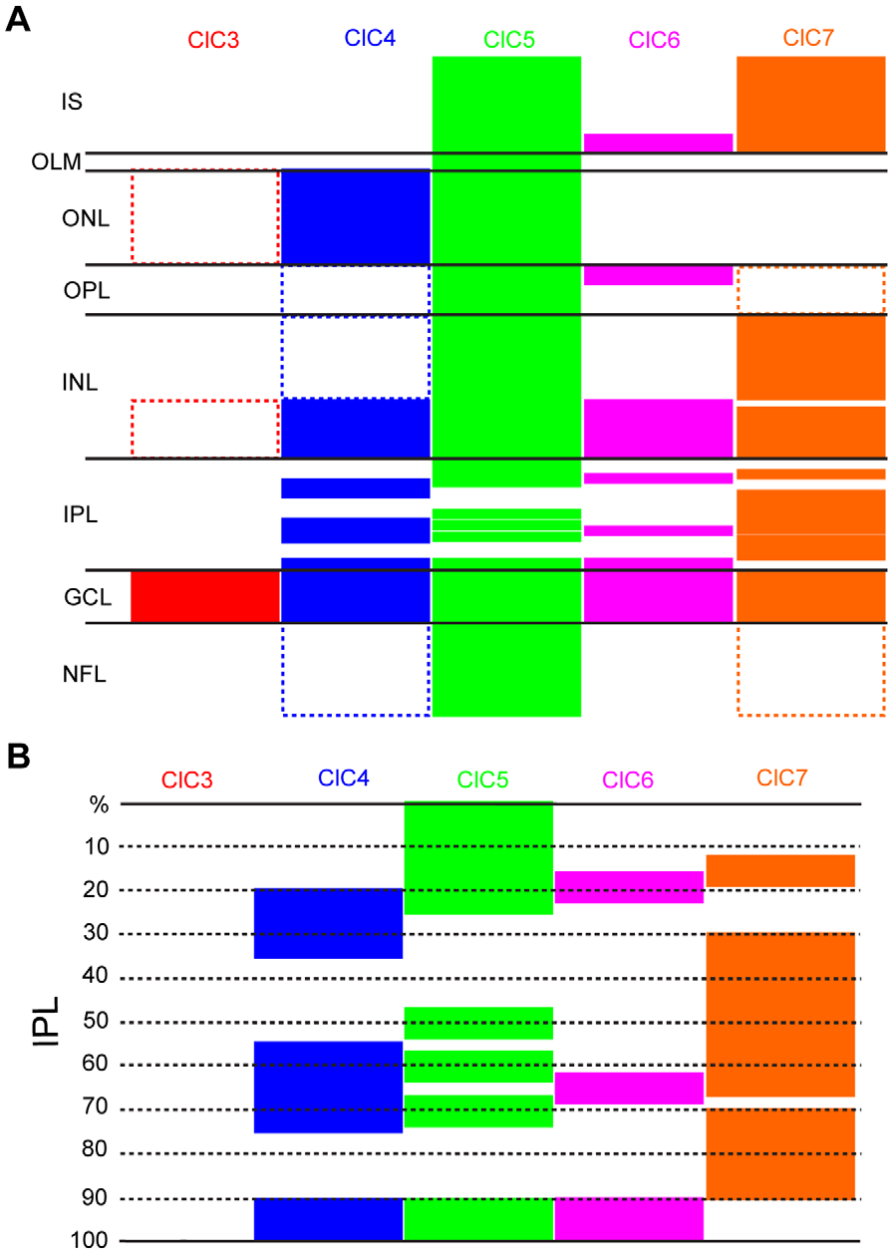
CLC H^+^/Cl^−^ antiporter expression in the chicken retina. **A**, Illustration of CLC transporter expression in the chicken retina. Filled blocks represent CLC expression. Unfilled blocks indicated with dashed lines represent weak CLC antibody labeling. **B**, CLC transporter expression in the inner plexiform layer.

Our investigation of CLC transporter expression revealed distinctive differences in the expression pattern of these proteins in the chicken retina (Fig. 12). Though there is evidence for CNS expression of all five CLC family members examined [15], [17], [20], [28], [50], this is the first study, to our knowledge, of relative CLC expression by different cell types within a single nervous system tissue.

### Retinal expression of ClC3 and ClC6

Our results suggest that ClC3 expression is weak and ClC6 expression is primarily localized to a restricted region of photoreceptors. This was a surprising result because ClC6 is known to be widely expressed in the nervous system [17] and ClC3 is necessary for normal retinal development [19] in mouse retina. In the case of ClC6, existing studies examining CNS expression of this protein did not look at retinal expression explicitly so it is possible that ClC6 distribution in the retina differs from that of other areas of the CNS. ClC3, however, has been shown to be expressed in synaptic layers of the mouse retina [19], [50] in contrast to our finding of low apparent ClC3 expression in chicken retina. It is possible that this difference reflects a true species difference. It is also possible that our ClC3 antibody did not have the same level of affinity for the protein. Arguing against this interpretation, a monoclonal antibody specific for a different region of the ClC3 protein also minimally labeled the chicken retina. Furthermore, this monoclonal antibody was raised against the same region of rat ClC3 as the polyclonal antibody that strongly labeled the synaptic layers of mouse retina in refs.19 and 50. The antigens used to synthesize both ClC3 antibodies were derived from portions of the rat protein sequence that are 100% identical to the predicted chicken amino acid sequence for ClC3.

### Diverse CLC expression patterns in photoreceptors

There is a high degree of variability in photoreceptor expression among ClCs 4–7. ClC7 is expressed in photoreceptor inner segments but is virtually absent from the ONL, whereas ClC4 is only expressed in the ONL. ClC5 is expressed throughout photoreceptors. Photoreceptor expression of ClC6 is restricted to synaptic terminals in the OPL and to vertical elements near the OLM. These vertical elements also appear to express ClC7 and could correspond to protein located near or within regions of the cilium and/or the ellipsoid of the photoreceptors. The different anatomical compartments of the vertebrate photoreceptor (outer segment, inner segment, and presynaptic) subserve different cellular functions (phototransduction, metabolism and gene expression, and synaptic transmission). Perhaps the variation in photoreceptor CLC expression is due to the specificity of different CLC transporters for the membranes of particular organelles. However, if that were solely the case, one would expect that ClC4 expression would be more similar to that of ClC5 because both localize to early endosomal compartments. Yet the expression of both ClC6 and ClC7 in vertical elements at the junction between the outer and inner segments may support a specific role for late endosomes and/or lysosomes at that location.

### ClC5 expression in the outer limiting membrane

The outer limiting membrane is a specialized retinal structure that serves as a semi-permeable boundary between the ONL and photoreceptor inner segments. It is not a membrane per se, but is composed of close appositions of the apical processes of Müller cells and photoreceptors. These appositions express proteins and structures characteristic of both tight junctions and adherens junctions and are thought to be important for maintaining retinal structure and potentially for regulating diffusion of material into the inner retina [51]. Though Müller cell and ONL expression of both ClC4 and ClC5 was pronounced, only ClC5 was expressed at the OLM. ClC4 and ClC5 are generally targeted to similar subcellular compartments. The presence of ClC5 (and not the closely related ClC4) in the OLM suggests a specific role for this transporter in OLM function.

### Retinal ClC7 expression is more restricted than ClC4 and ClC5

Our results show that ClC4 and ClC5 are fairly ubiquitously expressed in the nuclear and plexiform layers of the retina whereas the distribution of ClC7 is much more limited and specific. Calretinin-positive horizontal cells express ClC4 and ClC5, but not ClC7. In the chicken, all horizontal cells express either calretinin or TrkA, with calretinin-expressing cells comprising the majority [33]. We cannot rule out ClC7 expression in TrkA-positive horizontal cells because punctate ClC7 antibody label was present in the extreme outer portion of the INL, where horizontal cell bodies are located.

ClC7 expression in bipolar cells was more ambiguous. PKC is expressed by two populations of bipolar cells in the chicken retina [36], [52]. ClC4 and ClC5 were expressed in cell bodies and projections to the OPL from both populations. ClC4 and ClC5 signal in these cells was interior to that of PKC, supporting a cytosolic location for ClC4 and ClC5. Interestingly, though some PKC-expressing bipolar cells appeared to also express low levels of ClC7, the ClC7 antibody labeling co-localized with PKC on the margins of the cell body, consistent with plasma membrane localization. ClC7 expression is generally cytosolic, but there are examples of plasma membrane expression in some specialized cell types, such as osteoclasts [23].

In Müller cells, though there was some co-labeling by the ClC7 and glutamine synthetase antibodies, we could not discern whether this was due to close apposition of Müller processes to ClC7 puncta in neighboring amacrine cells or to true ClC7 expression in these retinal glial cells. Nonetheless, ClC7 signal was much less co localized with glutamine synthetase in comparison to ClC4 and ClC5, indicating that ClC7 expression by Müller cells, if any, is much less prevalent.

All CLC transporters examined were expressed in the inner third of the INL, consistent with expression by amacrine cells. Further examination of ClC4, 5, and 7 in double labeling experiments with the amacrine cell-specific marker, HPC-1 (syntaxin 1) confirmed expression of those transporters by amacrine cells. Strongly calretinin-positive amacrine cells expressed both ClC4 and ClC5, but not ClC7. The few amacrine cells that were PKC-positive were also positive for ClC4 and ClC5. Due to the highly punctate distribution of ClC7 antibody label, ClC7 expression by these cells was more ambiguous. More strikingly, ClC4 and ClC5 expression in amacrine cell bodies was highly co-localized with that of nNOS whereas very little overlap was seen between nNOS and ClC7. In contrast, ClC7 expression in ChAT-positive cells was very conspicuous. ClC5 was also expressed by cholinergic amacrine cells, but the co localization with ChAT was less prominent. Though this investigation of CLC transporter expression by different subtypes of amacrine cells is far from complete, it does suggest that these proteins may be preferentially expressed by amacrine cells that mediate different functions, especially in the case of ClC7. ClC4 and ClC5 appeared to be expressed in some degree by all amacrine cells whereas ClC7, despite some admitted uncertainty caused by the highly punctate nature of the signal, was clearly expressed in cholinergic amacrine cells. Whether this especially strong expression is based on an as yet un-described requirement for a greater number of lysosomes by starburst amacrine cells or on another unknown ClC7-mediated function is not clear at this time. However, it is interesting to note that other Cl^−^ transport proteins, NKCC and KCC cation-Cl^−^ co-transporters, have been found to be selectively expressed in different regions of cholinergic amacrine cells, where they actively maintain different local cytosolic Cl^−^ concentrations with important implications for cell function [53].

### CLC expression in ganglion cells

The ganglion cell layer is comprised of the cell bodies of ganglion cells and displaced amacrine cells. All CLC transporters examined were expressed in the GCL, and there was no great variation in their expression patterns in these cells. However, due to the persistence of ClC5 antibody signal in the GCL after pre-incubation with ClC5 peptide, we cannot rule out that some of the ClC5 labeling there is nonspecific. Interestingly, only ClC5 was strongly expressed in the NFL, and ClC5 antibody labeling there did not overlap with that of glutamine synthetase (Fig. 7O), suggesting that ClC5 is preferentially expressed in ganglion cell axons.

### CLC expression in the synaptic layers of the retina

ClC5 was widely expressed in the outer plexiform layer with expression located in the processes of horizontal cells, bipolar cells, and Müller cells. In contrast, ClC4 and ClC7 OPL expression was more diffuse, and individual ClC4 and ClC7 positive processes were rarely discernable. ClC4 antibody labeling in the OPL co-localized with PKC, but not with calretinin, suggesting that the transporter is expressed in bipolar cell postsynaptic terminals, but not in calretinin-expressing horizontal cell processes. ClC7 antibody labeling did not co-localize with either PKC or calretinin. ClC6 was moderately expressed in the OPL. The ClC6 signal in this layer showed a high degree of overlap with SV2, a specific marker of photoreceptor terminals. These observations suggest that the role of CLC transporters in signaling in the outer retina is most likely cell-type and transporter specific.

Another key locus for CLC transporter expression diversity in the chicken retina was the inner plexiform layer. All CLC transporters examined, except for ClC3, showed some degree of expression in both the ON and OFF sublamina of the IPL, and these expression patterns are summarized in Figure 12B. All four family members with discernable IPL expression were observed in both the inner and the outer regions of the IPL. ClC5 was the only CLC transporter with banding in the outermost section of the IPL (0–15% IPL depth), whereas ClC4 and ClC7 extended horizontal and vertical processes, respectively, and ClC6 was not expressed at all. In the innermost section of the IPL (90–100% IPL depth), an area thought to be particularly important for rod-driven signaling, antibodies specific for ClC4, 5, and 6 labeled a thin, well-defined PKC, nNOS, and calretinin immunoreactive band that was consistently negative for ClC7. In the central IPL, the expression of the different CLC transporters appeared to overlap to a higher degree. However, double label experiments with different markers revealed some subtle differences in their IPL expression patterns. IPL bands immunoreactive for ClC4 and ClC5 antibodies were also immunoreactive for calretinin, except at one calretinin-positive band at the center of the IPL. In contrast, ClC4 antibody co-localized with all PKC-positive IPL bands (but not bipolar cell terminals) in the outer IPL, whereas ClC5 only co-localized with PKC in the innermost immunoreactive IPL band. It is intriguing that ClC4 and ClC5 appeared to be expressed at dendrites but not terminals of bipolar cells, at least at the resolution level of light microscopy. Further examination of this apparent differential synaptic distribution at the electron microscope level would be of interest. ClC4 and ClC5 also co-localized with nNOS in the IPL although the overlap was less intense in the case of ClC4. ClC7 expression did not overlap with that of calretinin, PKC, or nNOS in the IPL, but the outermost band of ClC7 antibody immunoreactivity strongly co-localized with the outermost band of ChAT expression. ClC5 IPL expression overlapped with both bands of ChAT antibody labeling. Taken together, these observations suggest that the CLCs have synapse-specific functions, and it will be important to test their physiological functions in the context of synaptic transmission.

### Are CLCs differentially expressed in other tissues?

Most previous investigations of CLC transporter expression have focused on either organ level expression or organellar expression in various cell types. There has been comparatively little examination of the relative expression levels of various CLC proteins in different cell types, especially in the nervous system. Although there is evidence that all of the Cl^−^/H^+^ antiporters in the CLC protein family display similar biophysical properties and that they are fairly broadly expressed, they have been shown to mediate different functions. For example, knockout studies revealed that, in the kidney, ClC5 is essential for normal endocytosis in the proximal tubule whereas ClC7, also expressed in the proximal tubule, is necessary for normal lysosomal protein degradation [54]. Further, though both ClC3 and ClC7 gene disruption results in pronounced neurodegeneration, the neurodegenerative process is different for the two transporters [15].

It is likely that the observed differences in CLC transporter function stem at least in part from their different cellular addresses [55] and this localization pattern is fairly consistent across tissues. Little is known, however, about the functional significance of cell-type specific expression within a tissue. Jentsch and colleagues demonstrated a correlation between ClC5 expression and expression of the vacuolar proton pump in different kidney cell types [30]. Two splice variants of ClC4 are expressed in taste receptor cells, where they are hypothesized to mediate different functions, but are not expressed in neighboring taste-insensitive epithelial cells [56]. In the rat vas deferens and epididymus, ClC5 is located in narrow and clear cells with strong proton pump expression whereas ClC3 is expressed in principal cells with little proton pump expression, and ClC4 is not expressed at all [57]. The numerous knockout and expression studies done on the CLC Cl^−^/H^+^ exchangers have been extremely valuable in elucidating the biophysical characteristics of these transporters as well as in providing clues to their normal cellular function. The challenge remains, however, to examine the physiological role of these transporters in their native cellular context. The distinctive expression patterns of CLC Cl^−^/H^+^ exchangers in different retinal cell types and in the synaptic layers of the retina makes that endeavor compelling.

## Supporting Information

Figure S1 **Minimal ClC3 expression in chicken retina revealed by monoclonal antibody to ClC3 n-terminus.** **A**, Tissue section labeled only with secondary antibody. **B**, Retinal section labeled with monoclonal ClC3 antibody reveals a low level of labeling, if any. (Scale for A is 50 µm and applies for B as well.)
(TIF)

## References

[1] Jentsch TJ (2008). CLC chloride channels and transporters: from genes to protein structure, pathology and physiology.. Crit Rev Biochem Mol Biol.

[2] Smith AJ, Lippiat JD (2010). Direct endosomal acidification by the outwardly rectifying CLC-5 Cl(−)/H(+) exchanger.. J Physiol.

[3] Picollo A, Pusch M (2005). Chloride/proton antiporter activity of mammalian CLC proteins ClC-4 and ClC-5.. Nature.

[4] Scheel O, Zdebik AA, Lourdel S, Jentsch TJ (2005). Voltage-dependent electrogenic chloride/proton exchange by endosomal CLC proteins.. Nature.

[5] Graves AR, Curran PK, Smith CL, Mindell JA (2008). The Cl−/H+ antiporter ClC-7 is the primary chloride permeation pathway in lysosomes.. Nature.

[6] Matsuda JJ, Filali MS, Volk KA, Collins MM, Moreland JG (2008). Overexpression of CLC-3 in HEK293T cells yields novel currents that are pH dependent.. Am J Physiol Cell Physiol.

[7] Neagoe I, Stauber T, Fidzinski P, Bergsdorf EY, Jentsch TJ (2010). The late endosomal ClC-6 mediates proton/chloride countertransport in heterologous plasma membrane expression.. J Biol Chem.

[8] Steinberg BE, Huynh KK, Brodovitch A, Jabs S, Stauber T (2010). A cation counterflux supports lysosomal acidification.. J Cell Biol.

[9] Lorenz C, Pusch M, Jentsch TJ (1996). Heteromultimeric CLC chloride channels with novel properties.. Proc Natl Acad Sci U S A.

[10] Weinreich F, Jentsch TJ (2001). Pores formed by single subunits in mixed dimers of different CLC chloride channels.. J Biol Chem.

[11] Faundez V, Hartzell HC (2004). Intracellular chloride channels: determinants of function in the endosomal pathway.. Sci STKE.

[12] Novarino G, Weinert S, Rickheit G, Jentsch TJ (2010). Endosomal chloride-proton exchange rather than chloride conductance is crucial for renal endocytosis.. Science.

[13] Weinert S, Jabs S, Supanchart C, Schweizer M, Gimber N (2010). Lysosomal pathology and osteopetrosis upon loss of H+-driven lysosomal Cl− accumulation.. Science.

[14] Jentsch TJ, Neagoe I, Scheel O (2005). CLC chloride channels and transporters.. Curr Opin Neurobiol.

[15] Kasper D, Planells-Cases R, Fuhrmann JC, Scheel O, Zeitz O (2005). Loss of the chloride channel ClC-7 leads to lysosomal storage disease and neurodegeneration.. EMBO J.

[16] Kawasaki M, Uchida S, Monkawa T, Miyawaki A, Mikoshiba K (1994). Cloning and expression of a protein kinase C-regulated chloride channel abundantly expressed in rat brain neuronal cells.. Neuron.

[17] Poet M, Kornak U, Schweizer M, Zdebik AA, Scheel O (2006). Lysosomal storage disease upon disruption of the neuronal chloride transport protein ClC-6.. Proc Natl Acad Sci U S A.

[18] Steinmeyer K, Schwappach B, Bens M, Vandewalle A, Jentsch TJ (1995). Cloning and functional expression of rat CLC-5, a chloride channel related to kidney disease.. J Biol Chem.

[19] Stobrawa SM, Breiderhoff T, Takamori S, Engel D, Schweizer M (2001). Disruption of ClC-3, a chloride channel expressed on synaptic vesicles, leads to a loss of the hippocampus.. Neuron.

[20] Sakamoto H, Sado Y, Naito I, Kwon TH, Inoue S (1999). Cellular and subcellular immunolocalization of ClC-5 channel in mouse kidney: colocalization with H+-ATPase.. Am J Physiol.

[21] Lloyd SE, Pearce SH, Fisher SE, Steinmeyer K, Schwappach B (1996). A common molecular basis for three inherited kidney stone diseases.. Nature.

[22] Hara-Chikuma M, Wang Y, Guggino SE, Guggino WB, Verkman AS (2005). Impaired acidification in early endosomes of ClC-5 deficient proximal tubule.. Biochem Biophys Res Commun.

[23] Kornak U, Kasper D, Bosl MR, Kaiser E, Schweizer M (2001). Loss of the ClC-7 chloride channel leads to osteopetrosis in mice and man.. Cell.

[24] Hoffpauir B, McMains E, Gleason E (2006). Nitric oxide transiently converts synaptic inhibition to excitation in retinal amacrine cells.. J Neurophysiol.

[25] Fischer AJ, McKinnon LA, Nathanson NM, Stell WK (1998). Identification and localization of muscarinic acetylcholine receptors in the ocular tissues of the chick.. J Comp Neurol.

[26] Caldwell RB, Kierzek AM, Arakawa H, Bezzubov Y, Zaim J (2005). Full-length cDNAs from chicken bursal lymphocytes to facilitate gene function analysis.. Genome Biol.

[27] Suzuki T, Rai T, Hayama A, Sohara E, Suda S (2006). Intracellular localization of ClC chloride channels and their ability to form hetero-oligomers.. J Cell Physiol.

[28] Okkenhaug H, Weylandt KH, Carmena D, Wells DJ, Higgins CF (2006). The human ClC-4 protein, a member of the CLC chloride channel/transporter family, is localized to the endoplasmic reticulum by its N-terminus.. FASEB J.

[29] Luyckx VA, Goda FO, Mount DB, Nishio T, Hall A (1998). Intrarenal and subcellular localization of rat CLC5.. Am J Physiol.

[30] Gunther W, Luchow A, Cluzeaud F, Vandewalle A, Jentsch TJ (1998). ClC-5, the chloride channel mutated in Dent's disease, colocalizes with the proton pump in endocytotically active kidney cells.. Proc Natl Acad Sci U S A.

[31] Vandewalle A, Cluzeaud F, Peng KC, Bens M, Luchow A (2001). Tissue distribution and subcellular localization of the ClC-5 chloride channel in rat intestinal cells.. Am J Physiol Cell Physiol.

[32] Ellis JH, Richards DE, Rogers JH (1991). Calretinin and calbindin in the retina of the developing chick.. Cell Tissue Res.

[33] Fischer AJ, Stanke JJ, Aloisio G, Hoy H, Stell WK (2007). Heterogeneity of horizontal cells in the chicken retina.. J Comp Neurol.

[34] Koulen P, Brandstatter JH, Kroger S, Enz R, Bormann J (1997). Immunocytochemical localization of the GABA(C) receptor rho subunits in the cat, goldfish, and chicken retina.. J Comp Neurol.

[35] Caminos E, Velasco A, Jarrin M, Aijon J, Lara JM (1999). Protein kinase C-like immunoreactive cells in embryo and adult chicken retinas.. Brain Res Dev Brain Res.

[36] Fischer AJ, Seltner RL, Poon J, Stell WK (1998). Immunocytochemical characterization of quisqualic acid- and N-methyl-D-aspartate-induced excitotoxicity in the retina of chicks.. J Comp Neurol.

[37] Barnstable CJ, Hofstein R, Akagawa K (1985). A marker of early amacrine cell development in rat retina.. Brain Res.

[38] Sherry DM, Mitchell R, Standifer KM, du Plessis B (2006). Distribution of plasma membrane-associated syntaxins 1 through 4 indicates distinct trafficking functions in the synaptic layers of the mouse retina.. BMC Neurosci.

[39] Gleason E, Borges S, Wilson M (1993). Synaptic transmission between pairs of retinal amacrine cells in culture.. J Neurosci.

[40] Linser P, Moscona AA (1979). Induction of glutamine synthetase in embryonic neural retina: localization in Muller fibers and dependence on cell interactions.. Proc Natl Acad Sci U S A.

[41] Norenberg MD, Dutt K, Reif-Lehrer L (1980). Glutamine synthetase localization in cortisol-induced chick embryo retinas.. J Cell Biol.

[42] Prada FA, Quesada A, Dorado ME, Chmielewski C, Prada C (1998). Glutamine synthetase (GS) activity and spatial and temporal patterns of GS expression in the developing chick retina: relationship with synaptogenesis in the outer plexiform layer.. Glia.

[43] Fischer AJ, Stell WK (1999). Nitric oxide synthase-containing cells in the retina, pigmented epithelium, choroid, and sclera of the chick eye.. J Comp Neurol.

[44] Crousillac S, LeRouge M, Rankin M, Gleason E (2003). Immunolocalization of TRPC channel subunits 1 and 4 in the chicken retina.. Vis Neurosci.

[45] Bergmann M, Grabs D, Rager G (2000). Expression of presynaptic proteins is closely correlated with the chronotopic pattern of axons in the retinotectal system of the chick.. J Comp Neurol.

[46] Bergmann M, Grabs D, Roder J, Rager G, Jeromin A (2002). Differential expression of neuronal calcium sensor-1 in the developing chick retina.. J Comp Neurol.

[47] Sen M, Gleason E (2006). Immunolocalization of metabotropic glutamate receptors 1 and 5 in the synaptic layers of the chicken retina.. Vis Neurosci.

[48] Millar T, Ishimoto I, Johnson CD, Epstein ML, Chubb IW (1985). Cholinergic and acetylcholinesterase-containing neurons of the chicken retina.. Neurosci Lett.

[49] Hirano AA, Brandstatter JH, Brecha NC (2005). Cellular distribution and subcellular localization of molecular components of vesicular transmitter release in horizontal cells of rabbit retina.. J Comp Neurol.

[50] Maritzen T, Keating DJ, Neagoe I, Zdebik AA, Jentsch TJ (2008). Role of the vesicular chloride transporter ClC-3 in neuroendocrine tissue.. J Neurosci.

[51] Omri S, Omri B, Savoldelli M, Jonet L, Thillaye-Goldenberg B (2010). The outer limiting membrane (OLM) revisited: clinical implications.. Clin Ophthalmol.

[52] Negishi K, Kato S, Teranishi T (1988). Dopamine cells and rod bipolar cells contain protein kinase C-like immunoreactivity in some vertebrate retinas.. Neurosci Lett.

[53] Gavrikov KE, Nilson JE, Dmitriev AV, Zucker CL, Mangel SC (2006). Dendritic compartmentalization of chloride cotransporters underlies directional responses of starburst amacrine cells in retina.. Proc Natl Acad Sci U S A.

[54] Plans V, Rickheit G, Jentsch TJ (2009). Physiological roles of CLC Cl(−)/H (+) exchangers in renal proximal tubules.. Pflugers Arch.

[55] Stauber T, Jentsch TJ (2010). Sorting motifs of the endosomal/lysosomal CLC chloride transporters.. J Biol Chem.

[56] Huang L, Cao J, Wang H, Vo LA, Brand JG (2005). Identification and functional characterization of a voltage-gated chloride channel and its novel splice variant in taste bud cells.. J Biol Chem.

[57] Isnard-Bagnis C, Da Silva N, Beaulieu V, Yu AS, Brown D (2003). Detection of ClC-3 and ClC-5 in epididymal epithelium: immunofluorescence and RT-PCR after LCM.. Am J Physiol Cell Physiol.

