# Supplementary figures and images for: Expression and Localization of CLC Chloride Transport Proteins in the Avian Retina
Source: PLoS One. 2011 Mar 7;6(3):e17647. doi: 10.1371/journal.pone.0017647 (PMC3049779; doi:10.1371/journal.pone.0017647)

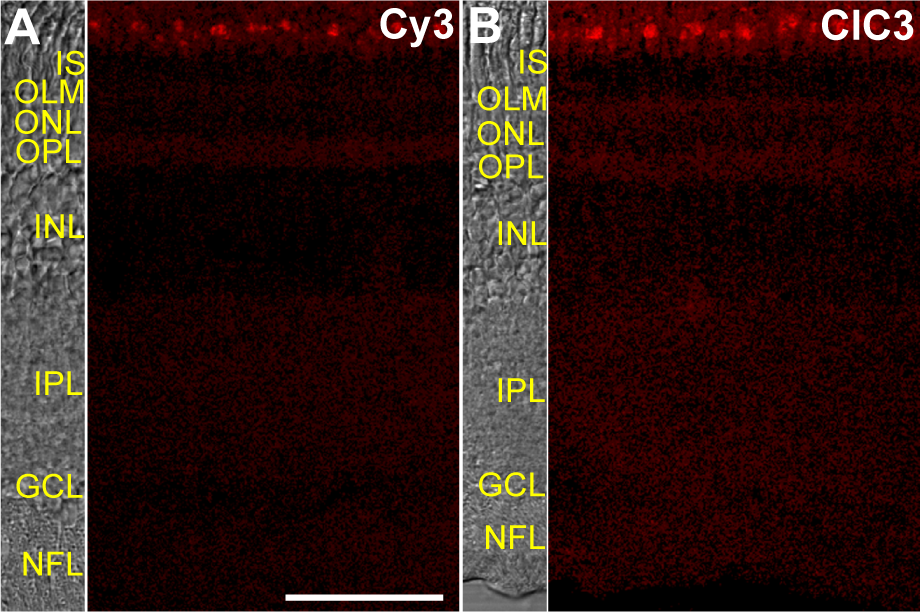

Supplement: Figure S1 — Minimal ClC3 expression in chicken retina revealed by monoclonal antibody to ClC3 n-terminus. A, Tissue section labeled only with secondary antibody. B, Retinal section labeled with monoclonal ClC3 antibody reveals a low level of labeling, if any. (Scale for A is 50 µm and applies for B as well.) (TIF) [file pone.0017647.s001.tif]
